# Single Assay for Simultaneous Detection and Differential Identification of Human and Avian Influenza Virus Types, Subtypes, and Emergent Variants

**DOI:** 10.1371/journal.pone.0008995

**Published:** 2010-02-03

**Authors:** David Metzgar, Christopher A. Myers, Kevin L. Russell, Dennis Faix, Patrick J. Blair, Jason Brown, Scott Vo, David E. Swayne, Colleen Thomas, David A. Stenger, Baochuan Lin, Anthony P. Malanoski, Zheng Wang, Kate M. Blaney, Nina C. Long, Joel M. Schnur, Magdi D. Saad, Lisa A. Borsuk, Agnieszka M. Lichanska, Matthew C. Lorence, Brian Weslowski, Klaus O. Schafer, Clark Tibbetts

**Affiliations:** 1 Department of Respiratory Diseases Research, Naval Health Research Center, San Diego, California, United States of America; 2 Division of the Armed Forces Health Surveillance Center, United States Department of Defense Global Emerging Infections Surveillance and Response System (GEIS), Silver Spring, Maryland, United States of America; 3 Southeast Poultry Research Laboratory, Agricultural Research Service, United States Department of Agriculture, Athens, Georgia, United States of America; 4 Naval Research Laboratory, Center for Bio/Molecular Science and Engineering, Washington, District of Columbia, United States of America; 5 George Mason University, Manassas, Virginia, United States of America; 6 Naval Medical Research Unit No. 3, Cairo, Egypt; 7 TessArae, LLC, Potomac Falls, Virginia, United States of America; Singapore Immunology Network, Singapore

## Abstract

For more than four decades the cause of most type A influenza virus infections of humans has been attributed to only two viral subtypes, A/H1N1 or A/H3N2. In contrast, avian and other vertebrate species are a reservoir of type A influenza virus genome diversity, hosting strains representing at least 120 of 144 combinations of 16 viral hemagglutinin and 9 viral neuraminidase subtypes. Viral genome segment reassortments and mutations emerging within this reservoir may spawn new influenza virus strains as imminent epidemic or pandemic threats to human health and poultry production. Traditional methods to detect and differentiate influenza virus subtypes are either time-consuming and labor-intensive (culture-based) or remarkably insensitive (antibody-based). Molecular diagnostic assays based upon reverse transcriptase-polymerase chain reaction (RT-PCR) have short assay cycle time, and high analytical sensitivity and specificity. However, none of these diagnostic tests determine viral gene nucleotide sequences to distinguish strains and variants of a detected pathogen from one specimen to the next. Decision-quality, strain- and variant-specific pathogen gene sequence information may be critical for public health, infection control, surveillance, epidemiology, or medical/veterinary treatment planning. The Resequencing Pathogen Microarray (RPM-Flu) is a robust, highly multiplexed and target gene sequencing-based alternative to both traditional culture- or biomarker-based diagnostic tests. RPM-Flu is a single, simultaneous differential diagnostic assay for all subtype combinations of type A influenza viruses and for 30 other viral and bacterial pathogens that may cause influenza-like illness. These other pathogen targets of RPM-Flu may co-infect and compound the morbidity and/or mortality of patients with influenza. The informative specificity of a single RPM-Flu test represents specimen-specific viral gene sequences as determinants of virus type, A/HN subtype, virulence, host-range, and resistance to antiviral agents.

## Introduction

There are sixteen recognized serological subtypes of type A influenza virus hemagglutinin (H1 through H16) and 9 type A neuraminidase subtypes (N1 through N9). Among the combinatorial diversity of 144 possible A/HN subtypes, relatively few subtypes have been identified as causes of human disease. Four pandemic outbreaks in the last century, one catastrophic, appear to have introduced the subsequently prevalent seasonal human influenza virus subtypes A/H1N1 (Spanish flu, 1918), A/H2N2 (Asian flu, 1957), A/H3N2 (Hong Kong flu, 1968), and A/H1N1 again (Swine flu, 1976; Russian flu, 1977). The current year 2009 has been marked by a late season pandemic-scale emergence of a novel A/H1N1 outbreak strain, raising immediate concerns for public health as well as for pork and poultry production industries worldwide.

As with the few common subtypes of human type A influenza viruses, there are similarly few subtypes of type A influenza viruses that are associated with most influenza infections of swine, horses or dogs. In distinct contrast, wildfowl species are natural hosts and a global reservoir for the majority of possible influenza A/HN subtypes. Many of these variant strains appear to be associated with endemic infections, often asymptomatic in avian hosts [1]. Incidental infections of humans by avian influenza viruses have been documented for avian influenza subtypes A/H5N1, A/H7N2, A/H7N3, A/H7N7, A/H9N2, A/H10N7 and A/H11N9. Recent outbreaks of “bird flu” may foreshadow an eventual pandemic outbreak, in the emergence of strains and variants with enhanced pathogenicity, virulence and transmissibility in human hosts. Examples of such outbreaks include A/H5N1 Hong Kong, 1997; H9N2 Hong Kong, 1999; A/H7N7 Netherlands, 2003; A/H5N1 Southeast Asia, 2004. Some avian A/H5 and A/H7 strains of influenza virus are recognized as highly pathogenic (HP) in domestic poultry and concerns arise that this phenotype may carry over to infections of humans. Since 1997, human infections associated with the Eurasian-African lineage of A/H5N1 HP avian influenza virus have been associated with 467 documented cases in 15 countries with high mortality (282 deaths) [2; updated 30 December 2009].

Fortunately, infectious transmission of such avian influenza virus strains between humans continues to be limited. However, history suggests that further evolution of these or other type A influenza strains could emerge as a next pandemic strain. Similarly, variant type A influenza virus strains have emerged from time to time, imposing serious costs and burdens upon poultry and livestock production.

Because the natural history and the molecular biology of influenza viruses reflect such viral genome diversity, there is a critical need for rapid, sensitive, specific, and informative assays to detect and characterize any subtype of influenza virus. Benchmark standard methods that employ propagation of virus in cell culture or in embryonating chicken eggs, with assays using panels of specific serological reagents, or reverse transcriptase polymerase chain reaction (RT-PCR)-based assays, using panels of short oligonucleotide primers and probes, are either slow and time consuming, or expensive. As prevailing strains of avian influenza continue to evolve and diverge, diagnostic assays that are based only on specific recognition of short signature sequences or peptide biomarker loci will increasingly fail, through false-positive and/or false-negative results. This will adversely impact critical decision-making.

This report describes a re-sequencing pathogen microarray (RPM)-based assay for simultaneous detection, identification and characterization of any subtype of type A human or avian influenza virus, based on rapid, sensitive and specimen-specific determination of nucleotide sequences from viral hemagglutinin, neuraminidase, and other genes.

## Methods

### Ethics Statement

All specimens described in this report that were originally obtained from human subjects were obtained with informed (verbal) consent of study participants allowing for further research use, following Institutional Review Board-approved research protocol NHRC.1999.0002, Triservice Population-Based Surveillance for Respiratory Pathogens Among High-Risk Military Personnel, CAPT Kevin Russell, MC, Principal Investigator. The NHRC IRB approved verbal consent for this protocol because of minimal risk to volunteers. Samples are collected for accredited diagnostic testing, and only used for further research in a de-identified manner with minimal demographic data (date and site of collection, age, sex), tested only for respiratory pathogens and analyzed in aggregate, and resulting data is not used for patient treatment or management. No part of this study was conducted as experimentation involving live vertebrate animals. Reference strains of avian influenza viruses were propagated in laboratory cultures for analysis of viral RNA.

### Specimen Collection and Sample Processing

The Naval Health Research Center (NHRC) collects, analyzes, and archives throat-swab specimens from human subjects as part of respiratory infection surveillance at US basic military training facilities. Research use of donated specimens is permitted under the local Institutional Review Board's approved protocol-compliant informed consent (see Acknowledgments). Throat swabs are suspended in stabilizing transport media and archived frozen at −80°C. Viral culture and PCR-based testing protocols are used at the NHRC diagnostic laboratory to determine the presence or absence of influenza virus, and these assays are accredited by the College of American Pathologists as compliant with the Clinical Laboratory Improvement Amendments of 1988, and the Department of Defense Clinical Laboratory Improvement Program of 1994.

Cloacal swabs and/or tracheal swabs from migratory birds (mostly waterfowl) and commercial poultry; tracheal and lung tissue samples from dead birds; human throat swabs and one human lung sample from a deceased patient were collected by Naval Medical Research Unit No. 3 (NAMRU-3, Cairo, Egypt). Avian influenza viruses in these samples were cultured using chicken eggs and/or Madin-Darby canine kidney cell cultures (MDCK, American Type Culture Collection, CCL-34). Sample collection and viral culture techniques were as described [3]. Total RNA was extracted and purified from culture isolates, and 1∶100 to 1∶1000 dilutions were used for diagnostic evaluations. Aliquots were forwarded from NHRC to the Naval Research Laboratory (NRL, Washington, DC) for RPM-Flu assay (TessArray RPM-Flu 3.1 Kit (RPM-Flu), TessArae, LLC, Potomac Falls, VA).

The U.S. Department of Agriculture, Agriculture Research Service (USDA-ARS) Southeast Poultry Research Laboratory (SEPRL, Athens, GA) selected specimens from its reference strain archive for blinded analysis by RPM-Flu. These samples comprised 20 representative avian influenza A/HN strains and also included 2 avian paramyxoviruses as controls. Total nucleic acid extractions and purifications (MagNA Pure, Roche Applied Science, Indianapolis, IN) were performed following harvest from infected eggs at SEPRL. A 30 µl aliquot from the final preparation was frozen and forwarded for RPM-Flu analysis.

### Reference Control Templates

The NRL group evaluated pathogen detection and identification capabilities of the RPM-Flu assay and kit components from TessArae, using control nucleic acid templates in assays *in lieu* of specimen total nucleic acid. Sources of control nucleic acid templates included type cultures of reference strain Eurasian-African A/H5N1 high pathogenicity influenza virus obtained from the Centers for Disease Control and Prevention (Atlanta, GA). Reference control templates as mixtures of the A/H1N1, A/H3N2, and B strains of influenza virus were from live virus preparations (Influenza Virus Vaccine Live, Intranasal, FluMist, 2004–2005 Formula; MedImmune Inc., Gaithersburg, MD), or from inactivated virus preparations (Influenza Virus Vaccine [Fluvirin] Purified Surface Antigen Vaccine, 2006–2007 Formula, Package Insert; Chiron Corporation/Novartis Vaccines and Diagnostics, Inc., Emeryville, CA). Synthetic DNA preparations were purchased from Blue Heron Biotechnology (Bothell, WA) as templates to represent other type A influenza hemagglutinin (H2, and H4 through H16) and neuraminidase (N3 through N9) gene sequences, as well as other pathogen gene sequences that are used as detectors on the RPM-Flu array. In some cases, the NRL group outsourced (Macrogen USA, Gaithersburg, MD) corroborative *de novo* gene sequence determinations from amplified products from hemagglutinin (HA), matrix (M), neuraminidase (NA), non-structural (NS1) and RNA-dependent RNA polymerase subunit (PB2) genes.

### RPM-Flu Assay

**Figure 1:**
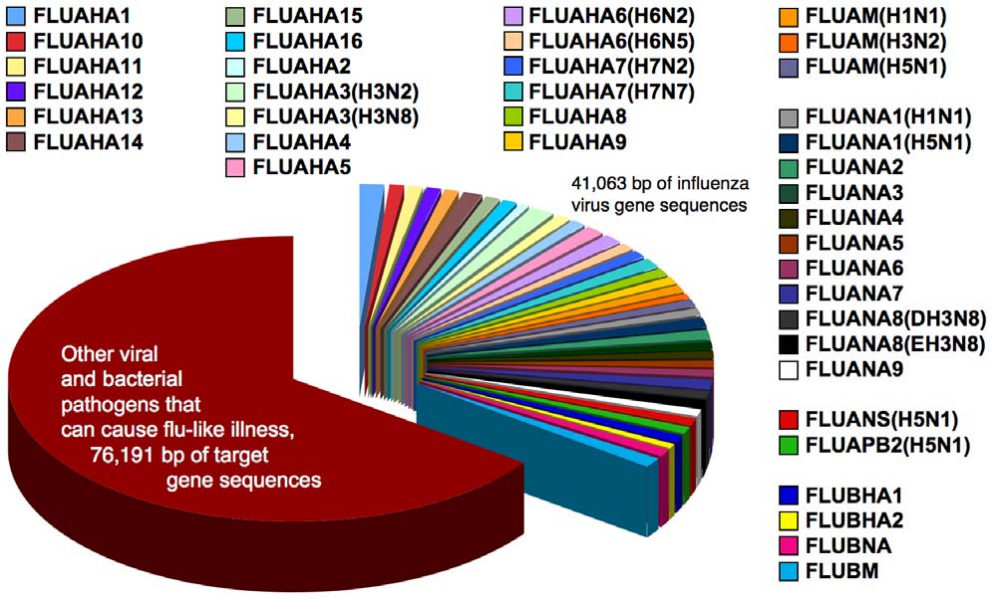
Influenza virus gene targets of the RPM-Flu assay. The TessArray RPM-Flu 3.1 allocates 40 detector tiles (41,063 bp) that represent influenza virus hemagglutinin genes (16 type A and 1 type B) and neuraminidase genes (9 type A and 1 type B), matrix genes (3 type A and 1 type B), and also the conserved NS and PB2 genes of avian influenza virus strain A/H5N1. The remaining 76,191 bp of re-sequencing capability on the microarray (as large red wedge) are distributed among 2 control detector tiles and 142 detector tiles that represent genes of other pathogens that may cause “flu-like” respiratory illness in humans. These other pathogens include viruses (adenovirus, enterovirus and rhinovirus, coronavirus, herpesvirus, measles virus, metapneumovirus, parainfluenzavirus, respiratory syncytial virus, rubella virus, and poxvirus [variola]) and bacteria (*Bacillus [anthracis]*, *Bordetella*, *Chlamydophila* and *Chlamydia*, *Corynebacterium*, *Francisella*, *Haemophilus*, *Klebsiella*, *Legionella*, *Moraxella*, *Mycobacterium*, *Mycoplasma*, *Neisseria*, *Pseudomonas*, *Streptococcus*, *Staphylococcus* and *Yersinia*).

Figure 1 illustrates the pathogen gene re-sequencing capacity of the RPM-Flu assay as both complementary strands of 117,254 bp of gene sequences, distributed across 188 detector tiles, representing 30 different categories of viral and bacterial respiratory pathogens. The 30 other types of viruses and bacterial targeted by the RPM-Flu assay as agents of flu-like illness are identified in Supplemental Information Figure S1. A single total nucleic acid preparation from a single aliquot of a single specimen is simultaneously assayed for possible detection and illumination of specimen-specific gene sequences from each of the 188 independent target pathogen gene sequencing detector tiles on the array.

The largest segment of the RPM-Flu microarray (41,063 bp) is allocated to 40 detector tiles to represent the multiple subtypes of type A and type B HA and NA genes, selected type A M genes, and conserved NS and PB2 gene sequences from avian influenza virus A/H5N1.

Selected specimens were thawed to retrieve aliquots for extraction and purification of total nucleic acid for multiple assays, including RPM-Flu testing. Each RPM-Flu assay consumed about 30 µl (2%) of the originally archived specimen (about 1.5 ml). RPM-Flu analysis of specimen total nucleic acid followed the manufacturer's recommendations (TessArae, LLC; protocol details are provided with the RPM-Flu 3.1 Kit User Manual and are available online at www.tessarae.com).

The RPM-Flu assay protocol is executed as a series of specimen and sample processing steps, listed below and indicating approximate time required for each step:

1. Extraction of total nucleic acid; reverse transcription to convert target RNA sequences to cDNA – 1 hour
2. Multiplexed amplification of targeted gene sequences by thermal cycling – 2.5 hour
3. Pooling, purification and fragmentation of amplification products – 0.5 hour
4. End-labeling (biotinylation) of amplification products – 0.5 hour
5. Hybridization of labeled products to microarrays – 4 hour to 16 hour (overnight preferred)
6. Washing and staining arrays – 1.5 hour
7. Scanning arrays for data acquisition – 0.15 hour

The abbreviated 4-hour hybridization time (5. above) reduces assay cycle time to single day, which may be helpful if same-day test results are a critical consideration. The overnight (∼16 hour) hybridization time is recommended and preferred to optimize both assay sensitivity and the quality and length(s) of assay-generated gene sequences from detected target pathogens.

### RPM-Flu Assay Data Analysis

Analysis of RPM-Flu assay data now differs from earlier reported through the development of the prototype assay platform RPMv1 [4], [5]. Each RPM-Flu assay of a specimen generates sequence data as base calls (A, G, C, or T) across each detector tile of Affymetrix CustomSeq-formatted microarrays [6] (see Affymetrix Technical Note, 2006, GeneChip CustomSeq Resequencing Array Base Calling Algorithm Version 2.0: Performance in Homozygous and Heterozygous SNP Detection). Image data acquired from GeneChip Instrument System scanning under GeneChip Operating Software control is translated by GSEQ sequence analysis software (Affymetrix Inc., Santa Clara, CA).

TessArray Sequence Analysis (TSEQ) software evaluates a “C3 Score” for each RPM-Flu detector tile, as a metric of detected DNA sequence quantity and quality. The C3 Score is the total number of GSEQ-identified nucleotides that appear in runs of three or more consecutive (non-N) base calls, expressed as percentage of the length (nucleotides) of each RPM-Flu detector tile sequence. This approach to definition of a detected sequence reduces background noise from spurious false-positive hybridizations of 25-base oligonucleotide probes that could lead to isolated and relatively uninformative single base calls.

Relaxed and stringent target pathogen detection and reporting thresholds for the RPM-Flu assay have been statistically and empirically established for all of the resequencing detector tiles at C3 Score >10 or C3 Score ≥20, respectively. RPM-Flu assay results from hundreds of clinical and field specimens as well as laboratory reference strains and synthetic DNA templates (aggregate data not shown) have demonstrated that these detection thresholds often represent C3 Scores that are more than 6 standard deviations (>6σ, *P*<0.000003) above the mean of C3 Scores from thousands of detector tiles from assays performed in the absence of positive control templates.

All sequences from an RPM-Flu assay that meet a detection and reporting threshold are automatically subjected to alignment-based search of the TessArray Validated Reference Sequence Database (VRSD) (most recently validated instance 10 August 2009), using a dedicated server-based implementation of the Basic Local Alignment Search Tool, BLAST, version 2.2.17 [7]. This enables identification of one or several most similar (and equivalently matching) sequence records for each target pathogen gene sequence that may be generated in the assay of every specimen. Adjustable parameters for the BLAST search are set as: word size 11 (or 7 if C3 Score <30), match/mismatch penalty +1/−1, gap costs as existence/extension +2/+1, low complexity region filter off, and mask options off.

In order to qualify for reporting of target pathogen detection using the relaxed threshold (C3 Score >10), a BLAST alignment and sequence similarity search using the assay-generated sequence of basecalls must return one or more most similar sequence records from the search database (as TessArae's VRSD) that are concordant with the identity of the tentatively identified target pathogen suggested by the identity of the particular resequencing detector tile.

### Viral Stocks for Determinations of Assay Sensitivity

Aliquots from two throat swab specimens collected during the 2008–2009 influenza were used for propagation of stocks by infection of MDCK cell cultures.

A/California-NHRC/BRD10622N/2009(H1N1)

A/California-NHRC/BRD10601N/2009(H3N2)

Infected culture lysates were then diluted for infections of replicate shell vial cultures to determine infectious titers (performed by ViraPur, San Diego, CA). The resulting stocks of BRD10622N(H1N1) and BRD10601(H3N2) were determined to contain 3.16×10^7^ TCID_50_/ml and 1.78×10^7^ TCID_50_/ml, respectively.

Serial ten-fold dilutions of viral stocks for RPM-Flu assays were prepared using fresh cell culture media as diluent, from 10^0^ (neat) to 10^−3^, and then three-fold dilutions were prepared from 1∶3,000 to 1∶2,187,000. The routine RPM-Flu protocol for total nucleic extraction and purification was performed on each of the triplicate diluted samples of the viral stocks for identity-blinded triplicate RPM-Flu assays.

## Results

### RPM-Flu Specificity: Analysis of Trivalent Influenza Vaccine

The influenza virus subtype A/H1 gene detector tile ‘HA1(H1N1)’ on the RPM-Flu microarray is an array of 11,808 resequencing oligonucleotide probes (25-mers), representing the nucleotides 113 through 1,588 of the A/New Caledonia/20/99 (H1N1) strain hemagglutinin gene (GenBank Accession Number AY289929). This is the actual A/H1N1 strain of influenza virus representing the viral HA1 gene product in the 2004–2005 vaccine configuration.

An aliquot of FluMist vaccine (2004–2005) was subjected to RPM-Flu assay, from which 1,351 specific base calls were determined across the HA1(H1N1) detector tile, resulting in C3 Score = 91.5. BLAST analysis returned AY289929 A/New Caledonia/20/99 (H1N1) as the most similar sequence record from alignment-based search across the entire GenBank, with the limit (minimum) expectation value of 0.0 (or E-value = 1.0e-180).

**Figure 2:**
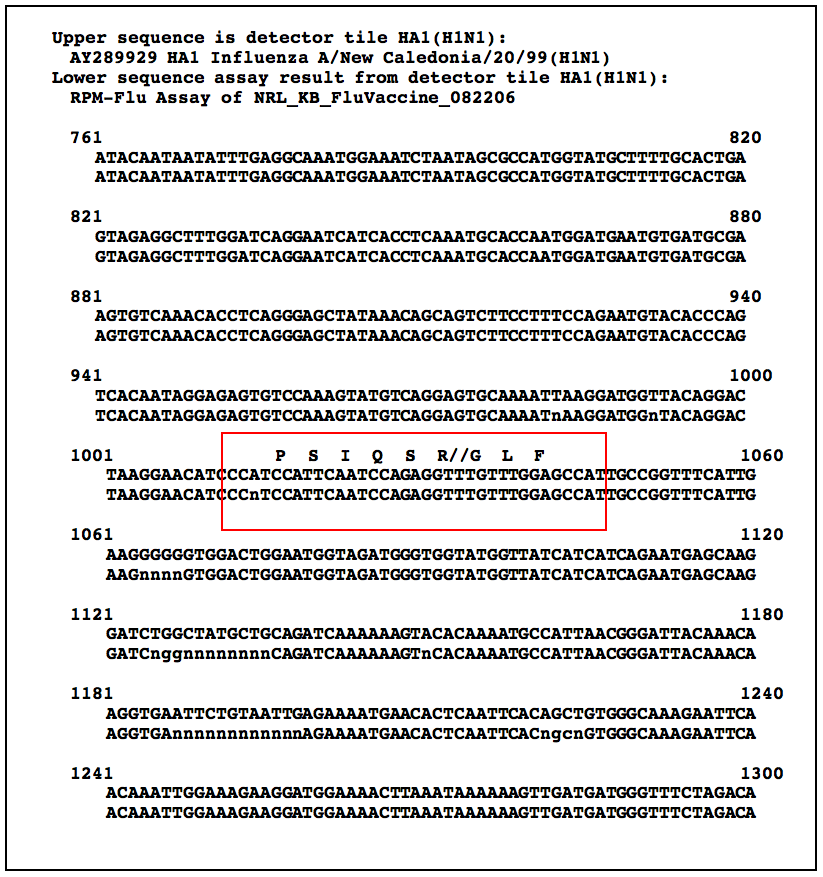
RPM-Flu assay-generated sequences from analysis of tri-valent influenza virus vaccine. A 540-nucleotide segment of the reported HA1 hemagglutinin sequence is shown aligned to the corresponding RPM-Flu HA1 gene detector tile sequence. This segment of the RPM-Flu HA1(H1N1) detector tile spans the locus of the A/H1 hemagglutinin peptide cleavage site (red box).

Figure 2 shows the alignment of a 540-nucleotide segment of this RPM-Flu assay detected sequence, below corresponding intervals of the RPM-Flu detector tile sequence. This part of the RPM-Flu HA1(H1N1) detector tile spans the locus of the A/H1 hemagglutinin peptide cleavage site [8]. Each of the nine successive 60 bp RPM-Flu assay-detected HA1 sequence segments shown in the inset was subjected to BLAST alignment and similarity analysis. These short query sequences (60 bp) are identical matches to corresponding hemagglutinin sequence segments of many different A/H1N1 isolates, but in each case, the list of identical sequence record(s) returned by BLAST includes one or more A/New Caledonia/20/99 (H1N1) records. This includes those 60 bp lines with runs of up to 12 uncalled bases (N). With the exception of small gaps of uncalled bases, the detected sequence is identical to the prototype sequence of the RPM-Flu HA1(H1N1) detector tile.

This example of the integrity of RPM-Flu assay-generated target pathogen gene sequencing for detection and identification is reinforced by results for the other specific hemagglutinin, neuraminidase, and matrix gene sequences from the same RPM-Flu assay of the same aliquot of FluMist vaccine. Table 1 shows that the most similar sequence records returned from BLAST analysis of assay-generated HA1, NA1, HA3, NA2, M(H1N1), M(H3N2) gene sequences, as well as type B HA, NA and M gene sequences, all correctly correspond to the actual influenza virus strains used to represent these components in the 2004–2005 vaccine configuration. This result is achieved even for instances (HA3, NA2, B-HA, B-NA and both the type A and type B M genes) where the RPM-Flu detector tile sequences and actual vaccine gene sequences do not precisely match. This reflects the robust capability of the RPM-Flu resequencing microarray to determine accurately the match or alternative mismatch of each target gene nucleotide relative to corresponding nucleotide of the detector sequence. These results also demonstrate the non-interference of similar assay targets for RPM-Flu detection and identification of multiple target gene sequences from the same specimen.

**Table 1:** Influenza type A and type B detector tile sequences and RPM-Flu assay-based identification of gene sequences by RPM-Flu assay of FluMist trivalent vaccine.

RPM-Flu detector title prototype sequences	C3 Score	E-value	SNPs^a^	Best matching sequence record (BLAST/GenBank)
Hemagglutinin genes				
A/New Caldedonia/20/1999 (H1N1)	64.0	1e-180	0/1039	A/New Caledonia/20/1999 (H1N1)
A/Canterbury/125/2005 (H3N2)	75.0	1e-180	6/1176	A/Wyoming/3/2003 (H3N2)
B/Malaysia/2506/2004	40.6	1e-155	10/451	B/Jilin/20/2003
B/Shanghai/361/2002	82.2	1e-180	6/750	B/Jilin/20/2003
Neuraminidase genes				
A/New Caldedonia/20/1999 (H1N1)	78.2	1e-180	0/971	A/New Caledonia/20/1999 (H1N1)
A/Canterbury/125/2005 (H3N2)	80.7	1e-180	7/999	A/Wyoming/3/2003 (H3N2)
B/Malaysia/2506/2004	61.7	1e-180	3/826	B/Jilin/20/2003^b^
Matrix genes				
A/Canterbury/100/2000 (H1N1)	69.5	1e-180	6/630	A/Ann Arbor/6/1960 (H2N2)
A/Canterbury/125/2005 (H3N2)	69.5	1e-180	14/626	A/Ann Arbor/6/1960 (H2N2)
B/Memphis/13/2003	81.6	1e-180	11/796	B/Ann Arbor/1/1966

a Number of observed single base polymorphisms (SNPs), as single base mismatches of detector and specimen gene sequences that are flanked by consensus base calls/number of specific base calls. The A/H1N1 detector tile and vaccine specimen hemagglutinin and neuraminidase gene sequences are concordant (bold font, underlined).

b The original RPM-Flu analysis on 21 Aug 2007 returned four best matching neuraminidase gene sequence records, as B/Yamagata/1246/2003, B/Taiwan/102/2005, B/Bangkok/460/2003, and B/Roma/4/2002. When subjected to BLAST analysis again on 09 Nov 2008, two more best matching neuraminidase gene sequence records were returned, as B/Taiwan/34/2004 and B/Jilin/20/2003. The actual influenza type B vaccine strain was B/Jilin/20/2003, but its neuraminidase gene sequence was not entered into the GenBank database until 21 Jul 2008.

Similarly specific typing, subtyping and strain identification results to those shown in Table 1 have been obtained from analyses of live and inactivated influenza vaccines configured for the 2005–2006, 2006–2007 and 2009–2010 seasons (results presented as SUPPLEMENTAL INFORMATION Tables S1, S2, S3 and S4). We note that results of assays using inactivated influenza virus vaccines identify detected type A matrix gene sequences are most closely (and correctly) matched by matrix gene sequence records from the A/Puerto Rico/8/34 (H1N1) master donor strain that is used for gene segment re-assortment to select for the inactivated vaccine strains.

The results in Table 1 are similar to those presented by Wang et al. [9, that report's Table 4]. That earlier work was performed using the RPMv1 prototype precursor platform of the TessArray RPM-Flu 3.1. The RPMv1 allocated only 7,691 base pairs (bp) to 9 different influenza-specific detector tiles, compared with the RPM-Flu assay that allocates 41,063 bp over 40 influenza virus-specific detector tiles (see Figure 1). The prototype RPMv1 assay also employed random primers in a reverse transcription and PCR-like protocol for total RNA amplification, and did not apply the C3 quantity/quality metric methodology described in this report for analysis of RPM-Flu assay-detected target pathogen gene sequences.

### RPM-Flu Specificity: Mutually Exclusive Detection and Identification of Different Subtypes of Human and Avian Influenza Viruses

Table 2 presents HA, NA and M gene detector tile C3 Scores from RPM-Flu assays of samples representing prevalent human subtypes A/H1N1, A/H3N2, and type B influenza viruses, inactivated trivalent influenza virus vaccine, and selected A/HN subtypes of type A avian influenza viruses. C3 Scores that failed to meet or exceed the RPM-Flu assay detection and reporting thresholds are shown in parentheses and in smaller font size to distinguish them from C3 Scores representing positive detection and identification of one or more of the RPM-Flu assay-targeted influenza viruses.

**Table 2:** Detection of specific HA, NA and M gene sequences from individual type A influenza virus reference strains or trivalent vaccine mixture of reference strains.

Template →	NHRC A/H1N1	NHRC A/H3N2	NHRC type B	Fluvirin vaccine	USDA A/H4N6	USDA A/H7N1	USDA A/H8N4	NAMRU A/H10N7	USDA A/H14N5
**HA Gene Detectors**									
HA1(H1N1) human	**76.5**	(4.3)	(1.9)	**91.4**	(1.2)	(1.9)	(0.8)	(1.4)	(1.0)
HA2(H3N2) human	(4.0)	(4.6)	(2.1)	(1.8)	(1.3)	(2.3)	(1.2)	(0.4)	(2.1)
FLUAHA3 human	(2.6)	**89.4**	(3.0)	**94.3**	(3.9)	(6.9)	(3.3)	(0.0)	(4.1)
FLUBHA human	(5.9)	(9.6)	**31.0**	**95.9**	(4.2)	(3.1)	(3.5)	(1.9)	(2.4)
FLUAHA3 avian	(3.5)	(7.0)	(2.9)	(8.4)	(4.9)	(4.4)	(1.8)	(0.0)	(3.3)
FLUAHA4 avian	(3.3)	(6.3)	(1.8)	(2.0)	**65.0**	(0.7)	(0.6)	(1.1)	(5.6)
HA5(H5N1) avian	(5.1)	(7.5)	(3.6)	(2.5)	(2.3)	(2.3)	(2.4)	(0.4)	(1.9)
FLUAHA6 avian	(3.3)	(5.1)	(1.6)	(2.3)	(1.3)	(1.5)	(2.2)	(1.4)	(1.6)
FLUAHA7 avian	(2.4)	(6.6)	(3.7)	(5.6)	(2.3)	**88.5**	(3.0)	(2.9)	(4.3)
FLUAHA8 avian	(3.3)	(2.8)	(1.6)	(3.0)	(0.9)	(1.6)	**76.4**	(0.0)	(1.4)
FLUAHA9 avian	(2.5)	(5.8)	(3.9)	(3.4)	(2.5)	(1.5)	(2.7)	(1.5)	(2.9)
HA1(H1N1)0 avian	(2.9)	(5.7)	(3.0)	(0.3)	(1.2)	(4.9)	(0.3)	**61.8**	(0.7)
HA1(H1N1)1 avian	(4.5)	(5.1)	(4.0)	(1.7)	(4.0)	(3.4)	(3.4)	(2.3)	(2.5)
HA1(H1N1)2 avian	(2.1)	(4.5)	(0.6)	(0.7)	(0.3)	(0.5)	(2.4)	(0.3)	(0.0)
HA1(H1N1)3 avian	(2.1)	(6.1)	(1.6)	(1.5)	(0.4)	(0.3)	(0.5)	(0.0)	(0.3)
HA1(H1N1)4 avian	(4.2)	(8.6)	(3.3)	(3.3)	(5.7)	(4.9)	(3.4)	(2.4)	**86.3**
HA1(H1N1)5 avian	(5.3)	(5.8)	(3.4)	(1.8)	(1.2)	(4.0)	(1.7)	(0.0)	(1.8)
HA1(H1N1)6 avian	(3.9)	(4.8)	(2.6)	(1.2)	(0.4)	(0.4)	(0.8)	(0.4)	(0.3)
**NA Gene Detectors**									
NA1(H1N1) human	**84.9**	(6.9)	(2.5)	**87.8**	(2.7)	**14.0**	(2.1)	(1.4)	(3.0)
NA2(H3N2) human	(4.7)	**84.9**	(1.5)	**96.8**	(2.1)	(3.2)	(0.9)	(0.7)	(0.7)
FLUBNA human	(2.8)	(4.8)	**13.7**	**98.0**	(0.8)	(2.0)	(1.7)	(0.9)	(2.7)
NA1(H1N1) avian	(8.0)	(8.3)	(1.8)	(6.1)	(2.5)	**57.5**	(3.9)	(1.0)	(5.9)
FLUANA3 avian	(3.8)	(5.3)	(3.1)	(0.8)	(1.3)	(1.9)	(1.1)	(0.3)	(2.9)
FLUANA4 avian	(3.2)	(4.4)	(2.5)	(2.5)	(3.1)	(9.8)	**67.7**	(0.0)	(5.5)
FLUANA5 avian	(5.4)	(9.8)	(4.4)	(2.9)	(4.4)	(5.2)	(4.7)	(1.5)	**66.3**
FLUANA6 avian	(6.4)	(7.9)	(3.9)	(5.3)	**85.5**	(8.6)	(5.4)	(1.8)	(7.4)
FLUANA7 avian	(5.5)	(7.7)	(4.4)	(3.3)	(3.7)	(4.9)	(3.0)	**23.1**	(3.0)
FLUANA8 avian	(5.9)	(7.6)	(4.4)	(6.1)	(6.6)	(5.0)	(5.6)	(1.1)	(5.2)
FLUANA9 avian	(4.7)	(6.9)	(1.6)	(1.5)	(3.8)	(3.7)	(2.9)	(0.0)	(1.7)
**M Gene Detectors**									
M(H1N1)	**88.7**	**51.0**	(2.8)	**61.6**	**50.1**	**50.8**	**40.4**	**44.5**	**66.3**
M(H3N2)	**60.6**	**96.5**	(2.1)	**59.2**	**45.0**	**46.2**	**48.1**	**42.4**	**48.6**
M(H5N1)	**38.1**	**39.1**	(4.8)	**37.0**	**51.2**	**69.6**	**45.6**	**44.1**	**39.2**
FLUBM	(4.3)	(5.2)	**34.3**	**97.7**	(2.6)	(2.7)	(2.9)	(2.3)	(2.7)

C3 Scores from RPM-Flu gene sequence detector tiles demonstrate specific and differential detection of human and avian influenza virus A/HN subtypes and human type B influenza virus in assays with single control templates, field or clinical specimens, or the three-part inactivated virus vaccine mix (A/H1N1, A/H3N2, and B). C3 Scores in smaller font, within parentheses, represent sub-threshold negative detector tile reports.

Except for the vaccine sample, assays of each sample reported sequences from only one hemagglutinin detector tile and one neuraminidase detector tile, those corresponding precisely to the specimen's A/HN subtype or B type.

The mean values of C3 Scores from the positive detector tiles in Table 2, and the background negative detector tiles, are respectively

Mean C3_pos_ = 73.7±24.0 (N = 22)

Mean C3_neg_ = 3.2±2.3 (N = 261)

[A six-sigma (6σ) detection threshold for this data set is C3 = 17.0]

The lowest positive control result from this data set was for the NA gene of the NHRC-type B influenza virus-positive specimen (C3_pos_ = 13.7). The same assay of this specimen generated higher C3 Scores for sequences from the B-HA (C3 = 31) and B-M (C3 = 34) detector tiles that exceeded the stringent detection threshold. The type B-NA sequence from the assay met the relaxed detection threshold (C3 Score >10), in part because BLAST analysis of the sequence returned nine most similar type B influenza virus NA gene sequence records. This corroborated a tentative detection report (relaxed threshold) that might have been made on the basis of the NA gene resequencing result alone. The specimen was collected in 2006 at U S Army Fort Leonard Wood, MO, and each of these most similar sequence records represented a NA gene from various circulating type B influenza virus strains collected during the same influenza season.

The RPM-Flu microarray includes four influenza virus matrix gene detector tiles, representing matrix gene sequences from selected reference strains of A/H1N1(human), A/H3N2(human), A/H5N1(avian) and B(human) types and subtypes. The results in Table 2 demonstrate specific discrimination and no apparent crosstalk between the three type A and the one type B matrix gene detectors. However for each of the human and avian subtypes of type A influenza viruses, it is generally the case that all three of the corresponding type A matrix gene detector tiles generate very similar and overlapping specimen-specific matrix gene sequences.

We have reviewed the lists of most similar sequence records returned by BLAST analysis of RPM-Flu assay-generated gene sequences from 12 different human (seasonal) A/H1N1 strains and 31 different human (seasonal) A/H3N2 strains.

Among RPM-Flu assay results from the 12 A/H1N1 samples there were 478, 800, 201, 348 and 312 most similar sequence records returned from BLAST analysis of sequences generated from the HA1(H1N1), NA1(H1N1), M(H1N1), M(H3N2), and NS(H5N1) detector tiles, respectively. Without exception, each of these most similar sequence records represents an A/H1N1 strain, including the 660 sequence records found to match the RPM-Flu sequences generated from the RPM-Flu M(H3N2) and NS(H5N1) detector tiles. Among the 31 A/H3N2 samples there were 754, 189, 1267, and 1678 most similar sequence records returned from the HA3(H3N2), NA2(H3N3), M(H1N1), and M(H3N2) detector tiles, respectively, and every one of these sequence records represents an A/H3N2 strain.

However, the NS (H5N1) detector tile generated 109 specimen-specific NS gene sequences from several of the A/H3N2 samples, and only 19 of the most NS gene sequence records were from A/H3N2 strains. The other NS gene sequence records that were found by BLAST to be most similar to the sequences generated from assays of A/H3N2 specimens represent subtypes A/H2N2 (12), A/H1N1 (73), A/H11N9 (2), A/H6N2 (2) and A/H3N6 (1). These results suggest that circulating strains of subtype A/H3N2 harbor diverse NS gene sequences, variations that presumably arise from frequent co-infections and genome segment reassortments in the field. On the other hand, the subtype A/H1N1 specimens led to RPM-Flu assay-generated NS gene sequences that are most similar to NS gene sequence records associated with subtype A/H1N1 strains.

Although it appears that the RPM-Flu assay-generated M gene sequences may have some utility to differentiate human A/H1N1 and human A/H3N2 subtypes, the NS gene sequences do not appear to be so reliable for the purpose of inferring A/HN subtype. This consideration will appear again later in RESULTS with the subject of indirect, inferred A/HN subtyping of type A avian influenza viruses.

### Clinical Sensitivity and Clinical Specificity of the RPM-Flu Assay for Human Type A Influenza Viruses

The RPM-Flu assay was implemented in a blinded analysis of several hundred clinical specimens from the respiratory infectious disease archives at NHRC. Selected throat swab specimens had been collected over the period 2005–2007 from basic military trainees at several installations in the United States. The subjects were evaluated at the time of specimen collection and associated with one of three cohorts: apparently healthy and afebrile individuals; those diagnosed with febrile respiratory illness (FRI); and those diagnosed with pneumonia (confirmed by chest x-ray).

A group of 298 specimens were analyzed using both the RPM-Flu assay and a validated benchmark RT-PCR assay for type A influenza virus described by Freed et al. [10]. Thirty specimens were identified as positive for type A influenza virus by both assays, and only 1 specimen was found to be negative by RPM-Flu and positive by RT-PCR. The remaining 267 specimens were found to be negative for influenza virus by both assays. All of the influenza-positive specimens were from participants that had been assigned to the FRI cohort.

Table 3 shows the calculation of high clinical sensitivity (97%) and even higher clinical specificity (100%) of RPM-Flu for detection and identification of type A human influenza virus, compared with results from testing the same specimens with the benchmark RT-PCR-based assay. Similarly high clinical sensitivity and high clinical specificity of RPM-Flu have been demonstrated with in the same ensemble of clinical specimens with respect to other simultaneously targeted pathogens of the assay, when similarly robust RT-PCR test panels were used for benchmark testing of matched specimens (e.g. human adenoviruses of subgroups B1, B2 and E; *Mycoplasma pneumoniae*; results not shown).

**Table 3:** Clinical sensitivity and clinical specificity of RPM-Flu for detection and identification of type A human influenza virus.

	RT-PCR Positive	RT-PCR Negative
RPM-Flu Positive	30	0
RPM-Flu Negative	1	267
Clinical Sensitivity = 96.8%±2.0% (95% confidence interval)		
Clinical Specificity = 100.0%±0.4% (95% confidence interval)		

RPM-Flu assay results for 298 clinical isolates are compared with results from benchmark RT-PCR test for type A influenza virus.

The benchmark RT-PCR type A influenza virus test used to assess clinical sensitivity and specificity of the RPM-Flu assay does not distinguish between the A/H1N1 and A/H3N2 subtypes. However the RPM-Flu assay is capable of simultaneous and mutually exclusive identification of a detected type A influenza virus as either seasonal subtype A/H1N1 or seasonal subtype A/H3N2. Results presented in Table 4 demonstrate this capability for the 30 specimens representing subtype A/H1N1 (5 specimens) or subtype A/H3N2 (25 specimens). Differentiation of the two prevalent subtypes is primarily based upon identification of assay-generated HA and NA sequences as matching HA1 or HA3 and/or NA1 or NA2, respectively. A secondary inference of A/HN subtype may be based on which of the M gene detectors M(H1N1) or M(H3N2) has the higher C3 Score. These indications of type A human influenza subtype are completely concordant with the corresponding subtypes associated with the most similar M gene sequence records from BLAST analysis of the sequences generated from both the M(H1N1) and the M(H3N2) detector tiles.

**Table 4:** RPM-Flu assay results C3 scores of six detector tiles for detection and identification of A/H1N1 and A/H3N2 influenza virus subtypes.

Detector tile →	HA1	HA3	NA1	NA2	M (H1N1)	M (H3N2)
A/H1N1-positive specimens						
NHRC_117	76.5		84.9		88.7	50.6
NHRC_051	35.4		42.1		75.0	31.1
NHRC_022	27.1		38.8		81.2	34.4
NHRC_157	14.4		14.7		15.2	
NHRC_010			13.2		14.1	
A/H3N2-positive specimens						
NHRC_044		89.4		84.8	51.0	96.5
NHRC_053		89.0		87.5	49.0	93.6
NHRC_396		78.1		79.7	43.5	91.9
NHRC_075		62.9		69.0	39.3	91.6
NHRC_154		73.0		6.8	37.1	90.8
NHRC_388		72.4		64.3	36.4	88.4
NHRC_189		59.0		59.5	35.7	87.1
NHRC_080		56.1		56.1	36.8	86.2
NHRC_385		60.3		61.2	32.1	84.1
NHRC_016		48.7		51.8	33.0	84.1
NHRC_376		59.8		43.7	32.8	81.9
NHRC_176		59.9		58.0	30.4	80.4
NHRC_393		60.3		44.7	22.2	58.6
NHRC_055		39.0		37.5	22.6	62.1
NHRC_156		38.8		24.6	15.3	42.4
NHRC_040		31.8		30.1		30.5
NHRC_382		36.9		17.0		24.5
NHRC_057		23.6			13.6	42.9
NHRC_204		13.5				27.9
NHRC_049		24.6				21.8
NHRC_378		22.3				11.3
NHRC_366		19.0				10.1
NHRC_026		22.7				
NHRC_390		18.2				
NHRC_380		14.2				

Each specimen was independently corroborated as positive for type A influenza virus by benchmark RT-PCR analysis, noting that that assay does not resolve the subtype. The A/H1N1- and A/H3N2-positive specimens identified from RPM-Flu results are listed in descending order relative to the sum of positive detector tile C3 Scores (for H, N, and M). Empty cells indicate detector tiles that did not meet the relaxed detection and reporting threshold (C3 Score <10).

In the course of increasing prevalence of oseltamivir-resistant strains of A/H1N1 and amantadine-resistant strains of A/H3N2, the NHRC has implemented a new RT-PCR panel to determine the A/H1 or A/H3 subtype for specimens already shown to be type A influenza virus-positive by the earlier RT-PCR panel (David Metzgar, Chris Myers, personal communication). They have also validated the assay by demonstrating concordance of subtyping results from new RT-PCR panel with a cell culture/hemagglutinin immunofluorescence assay (HAI). Type B influenza virus-positive specimens (identified from the first tier RT-PCR typing panel) were negative controls for the second tier RT-PCR-based Type A subtyping panel.

Twelve isolates from the 2008–2009 influenza season, six each testing positive for A/H1 or A/H3 by the RT-PCR panel (and also by culture-HAI), were selected for RPM-Flu analysis. All of the specimens tested definitively positive in the initial (and only necessary) RPM-Flu assay as either A/H1N1 or A/H3N2, matching the results of both the RT-PCR and HAI benchmark A/H1 and A/H3 assays. These results are summarized in Table 5, reinforcing the point of mutually exclusive detection and identification of specimen-specific gene sequences from the HA1 and NA1 detector tiles (as A/H1N1) or from the HA3 and NA2 detector tiles (as A/H3N2).

**Table 5:** Concordance of RPM-Flu determination of A/H1N1 and A/H3N2 subtypes with benchmark RT-PCR HA1/HA3 subtyping panel and cell culture-HAI HA1/HA3 subtyping assays.

	C3 Scores from RPM-Flu Detector Tiles					Diagnostic Assay		
Specimen (_2008)	HA1	NA1	M(H1N1):M(H3N2)	HA3	NA2	RPM-Flu	RT-PCR	Culture-HAI
NHRC_SV_418	72	71	87>48^a^	4	6	A/H1N1	A & H1	A & H1
NHRC_SV_413	64	71	81>46^a^	3	7	A/H1N1	A & H1	A & H1
NHRC_SV_415	35	42	75>31^a^	7	13	A/H1N1	A & H1	A & H1
NHRC_SV_416	8	12	20>10^a^	2	6	A/H1N1	A & H1	A & H1
NHRC_SV_411	15	22	35>17^a^	4	10	A/H1N1	A & H1	A & H1
NHRC_AML_10622	73	83	84>45^a^	0	0	A/H1N1	A & H1	A & H1
NHRC_JB_46159	2	0	15<56^b^	43	34	A/H3N2	A & H3	A & H3
NHRC_JB_30553	1	0	18<62^b^	45	34	A/H3N2	A & H3	A & H3
NHRC_JB_35105	1	1	3<17^b^	19	7	A/H3N2	A & H3	A & H3
NHRC_JB_65320	1	0	3<23^b^	18	7	A/H3N2	A & H3	A & H3
NHRC_JB_46162	1	1	21<64^b^	52	42	A/H3N2	A & H3	A & H3
NHRC_AML_10601	1	1	43<96^b^	80	79	A/H3N2	A & H3	A & H3

a BLAST analysis of RPM-Flu assay-generated sequences from both of these M gene detector tiles returned most similar M gene sequence records that exclusively represent subtype A/H1N1 strains.

b BLAST analysis of RPM-Flu assay-generated sequences from both of these M gene detector tiles returned most similar M gene sequence records that exclusively represent subtype A/H3N2 strains.

The results for M gene sequences from both the M(H1N1) and the M(H3N2) detector tiles for both sets of specimens shown in Table 5 again display significant crosstalk as overlapping and highly similar assay-generated sequences. In every example the two M gene sequences of an individual specimen, generated from the M(H1N1) and M(H3N2) gene detector tiles, are consistent with the A/HN subtype directly indicated the HA and NA gene sequences generated in the same RPM-Flu assay of the same specimen.

All 30 of the influenza-positive specimens described above were also reported by the individual RPM-Flu assays to be positive for at least one and up to six other viral and/or bacterial respiratory pathogens, including adenovirus, rhinovirus, coronavirus, *Moraxella catarrhalis*, *Staphylococcus aureus*, *Streptococcus agalactiae*, *Haemophilus influenzae*, *Neisseria meningitidis*, *Pseudomonas spp.*, *Streptococcus mitis*, and *Streptococcus pneumoniae*. There was no obvious correlation of the distributions of other co-infecting pathogens with the particular subtype of infecting influenza virus, or with particular specimen collection venue(s). There was no data on clinical presentation or outcomes to correlate with particular states of co-infection(s).

We considered whether or not there may be interference with the detection and identification of specific influenza viruses in specimens that test positive in RPM-Flu assays for other viral and bacterial respiratory pathogens. The background distribution of C3 Scores for detection and identification of influenza viruses was estimated from RPM-Flu assays of 14 different blank samples (water or transport media negative controls). The average C3 Score of the 39 type A and type B influenza virus detector tiles to be 1.7±1.1 (N = 14, results not shown).

Another set of 25 specimens were found to be negative for influenza virus by both RPM-Flu and benchmark RT-PCR tests, while the same RPM-Flu assays reported the specimens to be positive with apparently high load (C3 Score>75) of at least one other viral or bacterial respiratory pathogen (results not shown).

Simultaneous pathogen targets of the RPM-Flu assay that apparently do not interfere with detection and identification of influenza viruses include adenovirus (types Ad4, Ad7), coronavirus (strains OC43, 229E, NL63), rhinovirus (types A, B), metapneumovirus (types A, B), parainfluenzavirus (types 1, 2, 3), respiratory syncytial virus (RSV types A, B), *Chlamydophila pneumoniae*, *Haemophilus influenzae*, *Klebsiella pneumoniae*, *Moraxella catarrhalis*, *Mycoplasma pneumoniae*, *Neisseria meningitidis*, *Pseudomonas aeruginosa*, *Staphylococcus aureus* (including MRSA strains), *Streptococcus agalactiae*, *Streptococcus pneumoniae*, and *Streptococcus pyogenes*.

### Informational Specificity of RPM-Flu: Influenza Virus Genotypes and Resistance to Antiviral Agents

#### Subtype A/H3N2 influenza virus and Matrix (M) gene-based resistance to amantadine-like antiviral agents

The 25 A/H3N2-positive clinical specimens from the ensemble of 298 specimens described in the preceding section were collected between January 2007 and March 2007, at five different military basic training venues (Great Lakes Naval Training Station, IL; Marine Corps Recruit Depot, CA; Army Fort Leonard Wood, MO; Army Fort Jackson, SC; Army Fort Benning, GA). All but one of the RPM-Flu assay-generated HA3 gene sequences were most similar to BLAST-returned sequence records of A/H3N2 isolates that Nelson et al [11] associated with either major “clade a” from the 2006–2007 influenza outbreaks in the United States, or from the “N-lineage clade”, a lineage originating from the 2005–2006 influenza season and continuing to circulate nationwide during the 2007–2008 season. The HA3 gene sequence from a single A/H3N2 specimen was found to be most similar to sequence records that Nelson et al [11] associated with the 2006–2007 “minor clade b”. This minor clade b to represents a subpopulation of amantadine-sensitive A/H3N2 strains, in contrast to the resistant phenotype of the strains with the majority of circulating N- and a-clade strains.

At least five loci in the M2 trans-membrane peptide-encoding region of influenza virus M gene have been reported to confer some level of *in vitro* amantadine resistance [12], [13]. This suggested examination of the RPM assay-generated M gene sequences from these A/H3N2-positive specimens, to evaluate possible correlations with resistance of the subject isolates to amantadine.

**Figure 3:**
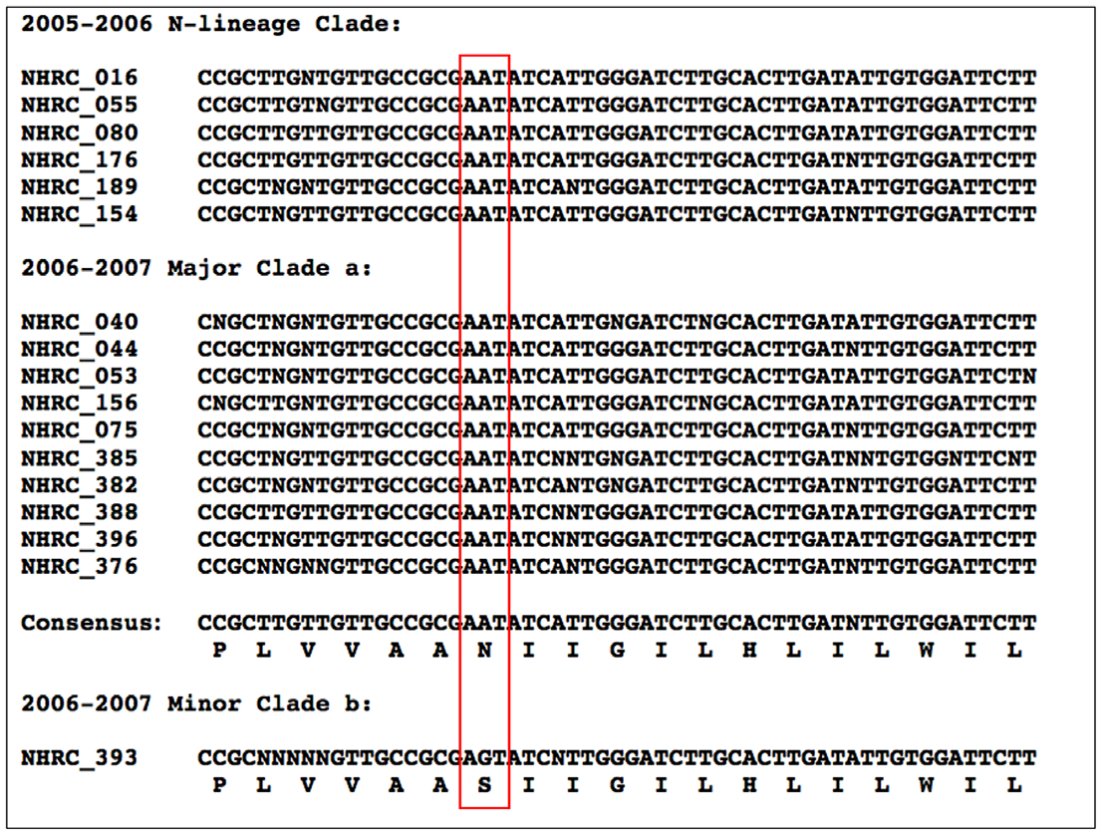
Alignment of M (matrix) gene sequences from 18 of 25 A/H3N2-positive specimens, representing three circulating 2006–2007 seasonal clades described by Nelson et al [11]. Twenty-four of the samples represent amantadine-resistant N-lineage or clade-a specimens, and each of these reveals -AAT- as asparagine (N) codon 31 of the M2 peptide (locus outlined in red box), a genotype consistent with the amantadine resistance phenotype. The matrix gene sequence of the single outlier clade-b sample (NHRC_393) reveals -AGT- as serine (S) codon 31 of the M2 peptide, a genotype consistent with the amantadine-sensitive phenotype.

Figure 3 shows alignment of M gene sequences from 18 of the 25 A/H3N2-positive specimens, together representing all three of the clades described by Nelson et al [11].

The M gene sequences detected by RPM-Flu assay of the 24 amantadine-resistant N-lineage or clade-a specimens all reveal -AAT- as asparagine (N) codon 31 of the M2 peptide, a genotype consistent with amantadine resistance. The matrix gene sequence of the outlier clade-b specimen (NHRC_393) reveals -AGT- as serine (S) codon 31 of the M2 peptide, a genotype consistent with an amantadine-sensitive phenotype.

#### Subtype A/H1N1 influenza virus and neuraminidase NA1 gene-based resistance to oseltamivir-like antivirals

The five A/H1N1-positive clinical specimens from the ensemble of 298 specimens described in the preceding section were collected between January 2007 and March 2007, at two different military basic training venues (Army Fort Jackson, SC; Army Fort Benning, GA). BLAST analysis of the five RPM-Flu-detected HA1 gene sequences returned most similar reference sequence records including those that Nelson et al. [11] associated with A/H1N1 Major Clade A from the 2006–2007 outbreaks of influenza in the United States. Global surveillance during the two most recent influenza seasons (2007–2009) has revealed that most circulating A/H1N1 isolates are now resistant to oseltamivir, compared with oseltamivir sensitivity of most if not all A/H1N1 isolates from the 2006–2007 influenza season [14], [15 (updated 13 June 2008)]. These more recent A/H1N1 strains appear to have a resistance phenotype due to mutation at the viral NA1 gene H275Y locus [16].

**Figure 4:**
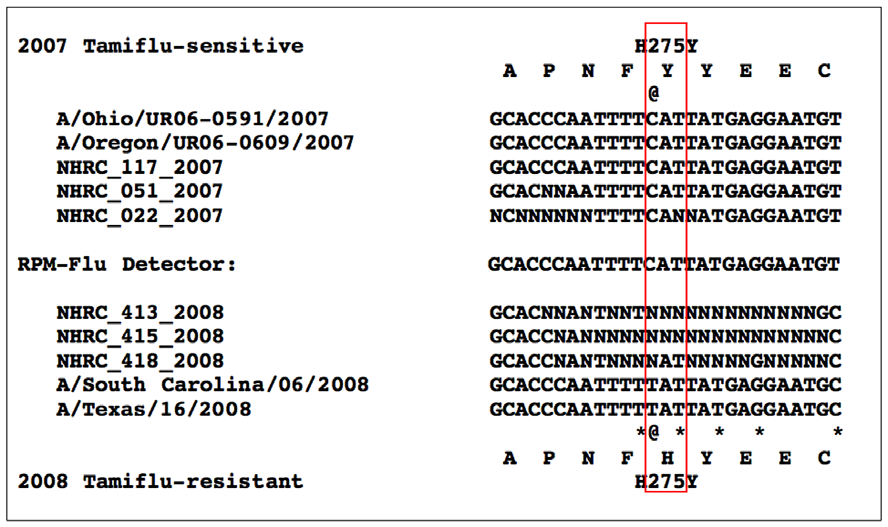
Comparison of RPM-Flu assay-generated NA1 (neuraminidase) gene sequences from two ensembles of clinical specimens, collected in 2006–2007 and in 2008, respectively. The older specimens have NA1 gene sequences that are concordant with those of the oseltamivir-sensitive, A/New Caldedonia/20/1999 (H1N1) RPM-Flu detector tile strain, including the determinant –CAT– histidine (H) codon at peptide position 275 (aligned locus in red box). BLAST analysis of these NA1 gene sequences returned most similar records including the oseltamivir-sensitive A/Ohio/UR06-0591/2007 and A/Oregon/UR06-0609/2007 strains. The ‘@’ symbol in the inset indicates the C-T transition mutations encoding tyrosine (Y) at this locus of more recent strains such as A/South Carolina/06/2008 and A/Texas/16/2008. Three specimens (NHRC_413, _415, and _418) were also collected in 2008, and these failed to generate sequence through this locus from the oseltamivir-sensitive detector tile genotype. These results do not explicitly corroborate the H275Y resistance genotype, although they are consistent with diminished re-sequencing efficiency at sites of mismatching detector tile and specimen gene sequences. These runs of N-calls suggest two or more proximal nucleotide mismatches may be present at this NA1 gene locus of the selected NHRC 2008 specimens.

Neuraminidase gene sequences were detected and compared at this locus from RPM-Flu assays of specimens from the earlier 2006–2007 season and more recent 2007–2008 season specimens provided by NHRC for analysis. As shown in the Figure 4, the RPM-Flu assay-generated NA1 gene sequences from the older ensemble of clinical specimens (2006–2007) were concordant with those of the oseltamivir-sensitive, A/New Caldedonia/20/1999 (H1N1) RPM-Flu detector tile strain. BLAST analysis of these NA1 gene sequences returned most similar records including the oseltamivir-sensitive A/Ohio/UR06-0591/2007 and A/Oregon/UR06-0609/2007 strains. The ‘@’ symbol in the inset indicates the C-T transition mutation corresponding to the H275Y mutation. On the other hand, the examples shown in the bottom half of the inset, representing 2007–2008 oseltamivir-resistant specimens from NHRC, had runs of 10 to 20 uncalled bases (N) spanning the NA1 gene's H275Y oseltamivir resistance locus. These results do not explicitly corroborate the H275Y resistance genotype, although they are consistent with diminished re-sequencing efficiency at sites of mismatching detector tile and specimen gene sequences. These runs of N-calls suggest two or more proximal nucleotide mismatches may be present at this NA1 gene locus of the selected NHRC 2008 specimens. There were 115 most similar sequence records returned by BLAST for the three full-length RPM-Flu assay-generated NA1 gene sequences, and five of these sequence records indicated a second, H275Y-proximal mutation (at positions of asterisks on either side of the @ symbol). Any such pair of proximal mismatches in a specimen gene sequence (template) could disrupt hybridization of the sets of 25-nucleotide oligomer probes used to determine basecalls by the RPM-Flu re-sequencing method.

Future iterations of the RPM-Flu microarray design could provide one or more alternative NA1 gene sequence detector subtiles. Such assay design iterations would enable future RPM-Flu assays to deliver more explicit first-assay characterization of the oseltamivir genotype and phenotype.

### RPM-Flu Sensitivity: Limits of Detection for A/H1N1 and A/H3N2 Isolates from the 2008–2009 Influenza Season

An isolate of subtype A/H1N1 and another of subtype A/H3N2 were selected by NHRC for single-passage propagation in MDCK cell cultures, followed by infectivity titrations in replicate shell vial cultures. The resulting neat stocks of BRD10622N(H1N1) and BRD10601(H3N2) were determined to contain 3.16×10^7^ TCID_50_/ml and 1.78×10^7^ TCID_50_/ml, respectively.

These two titered stocks were used to determine the analytical sensitivity of the RPM-Flu, by blinded assays of samples extracted from triplicate three-fold serial dilutions in the range of 1∶1,000 to 1∶2,187,000. Background C3 Scores were used as thresholds for reporting detection of either A/H1N1 or A/H3N2 in these limit dilution assays (as opposed to the relaxed and stringent thresholds used for reporting results from assays of clinical or field specimens). The background detector tiles represent 25 non-A/H1N1 and non-A/H3N2 detector tiles from each individual RPM-Flu assay, and a positive detection was reported if one of the target-specific detector tiles from the following lists had C3 score at least 3.29 standard deviations greater than the background mean C3 Score (p<0.001).

**Figure 5:**
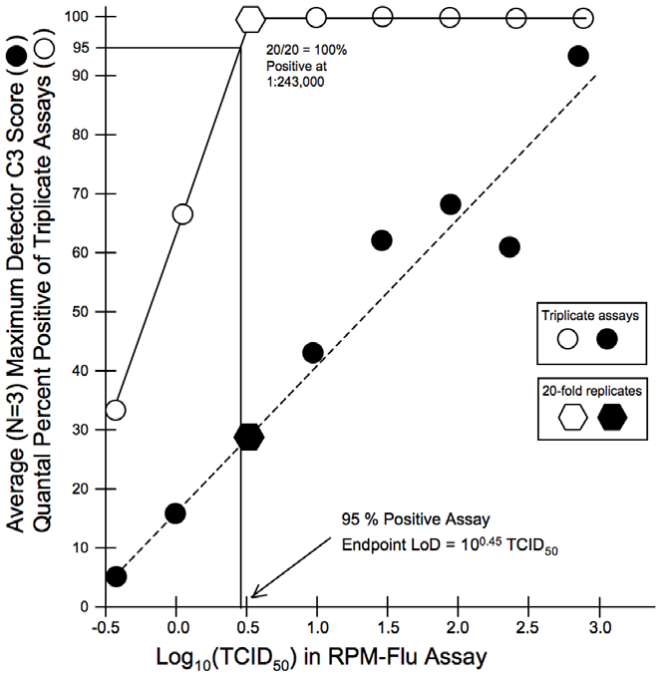
Limit of Detection (LoD) determination for seasonal A/H1N1 strain BRD10622. Neat stock of cell culture lysate (3.16×10̂7 TCID_50_/ml) was diluted 1∶1,000 in cell culture media. Triplicates at this dilution, and at serial three-fold dilutions to 1∶2,187,000 were individually extracted and subjected to RPM-Flu assay. Black circles indicate average of the maximum C3 Score (from either HA1, NA1 or M(H1N1) detector tiles) at each dilution, plotted against log_10_ TCID_50_ input per assay. Open circles represent the quantal percentage of positive assays (out of three) at each dilution tier. The open and black hexagons correspond to a dilution tier at which 20-fold replicates were assayed to demonstrate the 95% endpoint LoD, in this case corresponding to single assay input of about 3 TCID_50_.

**Figure 6:**
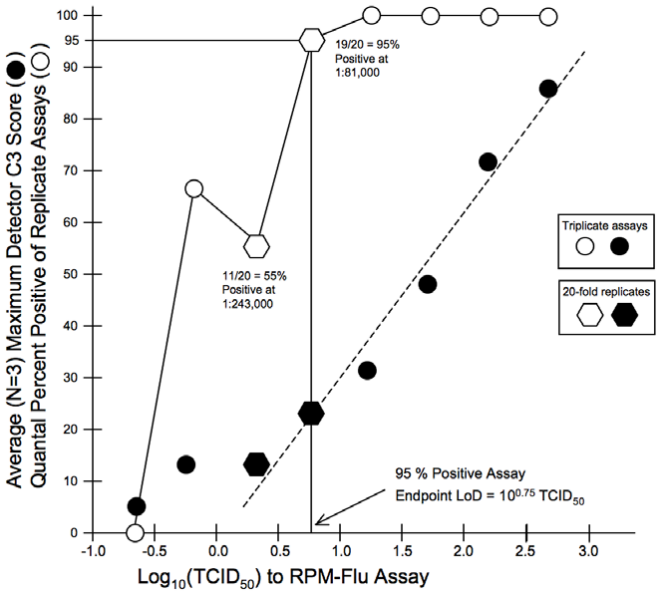
Limit of Detection (LoD) determination for seasonal A/H3N2 strain BRD10601. Neat stock of cell culture lysate (1.78×10̂7 TCID_50_/ml) was diluted 1∶1,000 in cell culture media. Triplicates at this dilution, and at serial three-fold dilutions to 1∶2,187,000 were individually extracted and subjected to RPM-Flu assay. Black circles indicate average of the maximum C3 Score (from either HA3, NA2 or M(H3N2) detector tiles) at each dilution, plotted against log_10_ TCID_50_ input per assay. Open circles represent the quantal percentage of positive assays (out of three) at each dilution tier. The open and black hexagons correspond to a dilution tier at which 20-fold replicates were assayed to demonstrate the 95% endpoint LoD, in this case corresponding to assay input of about 6 TCID_50_.

Figure 5 and Figure 6 illustrate the results of these endpoint dilution titrations of the titered A/H1N1 and titered A/H3N2 stocks, respectively, plotting the average highest C3 Score of A/H1N1 or A/H3N2 detector tiles for each of the triplicate assays at each dilution level. The percent positive assays (3/3, 2/3, 1/3 and/or 0/3) are also shown for the A/H1N1triplicate dilutions (Figure 5) and for the A/H3N2 triplicate dilutions (Figure 6). These unblinded dilution titration assay results were used to estimate 95% positive detection endpoints. Then one of the A/H1N1 and two of the A/H3N2 dilution tiers were expanded from three to twenty replicates, in order to demonstrate the high likelihood (≥95%) of positive detection and identification at the indicated dilution of the respective neat stocks.

From these results RPM-Flu assay, the 95% positive endpoint dilution limits of detection (LoD) be 3 TCID_50_ for the A/H1N1 titered stock preparation and 6 TCID_50_ for the A/ H3N2 titered stock preparation.

From the total of 41 independently extracted dilution specimens of the subtype-confirmed and titered A/H1N1 stock (Figure 5), 37 were reported to be influenza-positive by RPM-Flu assay, and all of these assays correctly reported by the RPM-Flu assays as subtype A/H1N1 (100% specificity). From a total of 58 independently extracted dilution specimens of the subtype-confirmed and titered A/H3N2 stock (Figure 6), 44 were reported to be influenza-positive by RPM-Flu assay, and all of these assays correctly reported by the RPM-Flu assays as subtype A/H3N2 (100% specificity). A total of 268 specimens from the NHRC clinical specimen ensemble were found to be negative for type A influenza virus by the RPM-Flu assay and 267 of these were also reported to be influenza virus-negative by benchmark RT-PCR assays for type A influenza virus, supporting the conclusion of 100% subtyping specificity for the RPM-Flu test.

### RPM-Flu Specificity: Direct Differentiation of Diverse Type A Avian Influenza Virus Subtypes Based upon RPM-Flu Assay-Generated HA and NA Gene Sequences

A collection of 23 reference strain specimens, representing diverse type A avian influenza virus subtypes, were propagated in embryonated eggs by SEPRL, then extracted for purification of total nucleic acid as input to RPM-Flu assays.

Table 6 presents the results for 19 of these 23 specimens reported by the RPM-Flu assays to be positive for avian influenza virus. In each case the RPM-Flu assay-generated HA and NA gene sequences identified the particular A/HN subtype that precisely matched the unblinded identifications of each reference strain. BLAST/GenBank analysis of these RPM-detected query sequences returned best matching sequence records with the same A/HN types as determined from the HA and NA detector tile C3 Scores. For 14 of these specimens, the single or multiple most similar HA and NA gene sequence records included the particular reference strain actually provided by SEPRL for analysis. These instances are highlighted as entries with flanking asterisks in the right column of Table 6).

**Table 6:** RPM-Flu Assay detection and subtype identification of avian influenza in reference strain specimens provided by SEPRL.

Blinded sample ID	Highest HA C3 Score	Highest NA C3 Score	RPM-Flu Detection & Identification	Unblinded SEPRL Reference Strain
**USDA_1**	**36.6**	**14.7**	**A/H1N1**	* A/Turkey/Kansas/4880/80 (H1N1) *
**USDA_2**	**30.0**	**85.3**	**A/H2N8**	* A/Herring Gull/DE/677/88 (H2N8) *
**USDA_3**	**65.6**	**78.0**	**A/H3N2**	**A/turkey/MN/366767/2005 (H3N2)**
**USDA_4**	**65.0**	**85.5**	**A/H4N6**	* A/Blue Winged Teal/OH/240B/88 (H4N6) *
**USDA_5**	6.7	4.6	**Negative for avian influenza virus**	**No avian influenza virus, Attributed to inoculum failure**
**USDA_6**	**12.1**	**18.6**	**A/H7N2**	* A/quail/PA/20304/98 (H7N2) *
**USDA_7**	**76.4**	**67.7**	**A/H8N4**	**A/turkey/CO/169118-13/02 (H8N4)**
**USDA_8**	**11.2**	**88.7**	**A/H11N9**	**Original inoculum as A/chicken/NJ/12220/97(H9N2) was found to be coinfected with A/H11N9 strainA/H11 sequence confirmed by DNA sequencingNA sequence pending**
**USDA_9**	**39.3**	**8.8**	**A/H10N7**	**A/quail/NJ/25254-22/95 (H10N7)**
**USDA_10**	**12.0**	**69.8**	**A/H11N3**	**A/chicken/NJ/4645/96(H11N3)**
**USDA_11**	**74.0**	**9.5**	**A/H12N5**	* A/duck/LA/188D/87 (H12N5) *
**USDA_12**	**16.8**	**18.0**	**A/H13N6**	* A/Gull/Maryland/1824/1978(H13N6) *
**USDA_13**	9.8	4.9	**Negative for avian influenza virus**	**No avian influenza virusAvian metapneumovirus (Colorado strain)**
			**Positive for avian metapneumovirus**	****
**USDA_14**	**64.4**	**22.8**	**A/H5N3**	* A/duck/Singapore/F119/97 (H5N3) *
**USDA_15**	**10.8**	**30.3**	**A/H7N3**	* A/chicken/Chile(F0)/176822/02 (H7N3) *
**USDA_16**	9.8	6.4	**Negative for avian influenza virus**	**No avian influenza virusAvian paramyxovirus type 1Newcastle Disease Virus California/02**
			**Positive for NDV using RPM-TEI**	****
**USDA_17**	**10.5**	**19.2**	**A/H5N2**	* A/chicken/Mex/26654-1374/94 (H5N2) *
**USDA_18**	**88.5**	**57.5**	**A/H7N1**	* A/turkey/Italy/4580/99 (H7N1) *
**USDA_19**	**66.3**	**26.1**	**A/H7N3**	* A/chicken/Pakistan/1369-CR2/95 (H7N3) *
**USDA_20**	**27.6**	**24.1**	**A/H7N7**	* A/chicken/Victoria/85 (H7N7) *
**USDA_21**	**86.3**	**66.2**	**A/H14N5**	* A/mallard/Gurjev/263/82 (H14N5) *
**USDA_22**	**80.9**	**17.9**	**A/H15N9**	* A/Shearwater/W. Australia/2576/79 (H15N9) *
**USDA_23**	10.4	**57.0**	**A/H?/N4**	**No detection of HA sequence.Best matching record forNA4 sequence is A/H8N4.**

For 14 of these specimens, the single or multiple most similar HA and NA gene sequence records matching sequences from the RPM-Flu assay included the particular reference strain actually provided by SEPRL for analysis. These instances are with asterisks flanking entries in the right column of Table 6). Other instances, without asterisks, represent assayed reference strains for which one or both HA and NA gene sequences had not yet been deposited in GenBank, but which were nevertheless concordant with sequences generated by the RPM-Flu assay.

The RPM-Flu assay of specimen USDA_13 was reported negative for detection of avian influenza virus, but the same assay also reported the specimen to be positive for avian metapneumovirus. This was revealed to be a correct result after unblinding of the specimen identification as a non-influenza control Type C avian metapneumovirus.

Three avian influenza virus-negative RPM-Flu assay results were also concordant with subsequently unblinded specimen information. USDA_5 was RPM-Flu negative for avian influenza and later confirmed to have been from an unsuccessful inoculation of the egg culture.

USDA_8 was reported by RPM-Flu assay to be A/H11N9, and while the initial identification from SEPRL was subtype A/H9N2, the original specimen apparently represented a mixed infection of H11N9 and H9N2. The HA component of the specimen was subjected to *de novo* gene sequencing and determined to be A/H11 strain. The RPM-detected neuraminidase sequence (868 bp, C3 = 88.7) unequivocally matched A/N9; the mean C3 Score of all other neuraminidase detector tiles in the assay was 3.1±1.6 (N = 10).

USDA_16 was correctly negative by RPM-Flu assay for avian influenza, but unblinding revealed this specimen to be a second negative control, containing instead avian paramyxovirus type I (Newcastle disease virus). After receiving this information, another aliquot of the same nucleic acid preparation from specimen USDA_16 was tested using an alternative RPM-TEI assay [17] for detecting tropical and emerging infections. This assay resulted in successful detection and identification of avian paramyxovirus type 1.

Specimen USDA_23 was received later as a substitute for the failed inoculum specimen USDA_5. The RPM-Flu assay of this specimen resulted in detection of sequence for neuraminidase type N4, with most similar sequence record representing an A/H8N4 strain. However, no hemagglutinin gene sequences were detected that met even the relaxed detection threshold. The highest C3 Scores among all RPM-Flu hemagglutinin detector tiles were HA1(H1N1-1918), a very short detector tile, and FLUAHA7, but neither of these sub-threshold results triggered a positive detection report.

Lin et al (2009) recently reported another study of avian influenza viruses using RPM-Flu assays for 24 AH5N1-positive field and clinical specimens collected by the NAMRU-3 group from infected birds and humans [18]. Supplemental Table S5 summarizes results from our re-analysis of their original RPM-Flu assay data (GSEQ FASTA files), presenting C3 Scores as described in this report for assay-generated sequences from the subtype-specific HA and NA detector tiles, or from the RPM-Flu M, NS and PB2 detector tiles (that represent conserved gene sequences of a prototype A/H5N1 strain).

The RPM-Flu assay-generated HA5 gene sequences of all the A/H5N1-positive specimens from NAMRU-3 and also of the A/H5N2 reference strain specimen from SEPRL (Table 6, USDA_17) were consistent with an extended arginine-rich and lysine-rich motif spanning the HA5 peptide cleavage site, consistent with a viral high pathogenicity (HP) phenotype [19], [20]. BLAST analysis of the RPM-Flu detected HA5 gene sequences from these strains returned most similar sequence records encoding a similar HP-like HA5 cleavage site motif.

The hemagglutinin RPM-Flu assay-generated HA5 gene sequence for the A/H5N3 reference strain (Table 6, USDA_14) had a significant gap of uncalled bases at the HA cleavage site locus, compared to the HP-like HA gene detector tile sequence A/Viet Nam/1203/2004 (H5N1). The most similar hemagglutinin and neuraminidase gene sequence records returned by BLAST for HA and NA gene sequences of USDA_14 included strain A/duck/Singapore/3/1997 (H5N3), the actual reference strain submitted (blinded) for RPM-Flu analysis. The encoded hemagglutinin gene cleavage site for this reference A/H5N3 strain is consistent with a low pathogenicity (LP) phenotype.

A/duck/Singapore/3/1997(H5N3) -PQRE----TR/GLF- (LP)

A/Viet Nam/1203/2004 (H5N1) -PQRERRRKKR/GLF- (HP)

These results demonstrate efficacy of RPM-Flu for unequivocal diagnostic detection and identification of human and avian influenza virus subtypes, based upon direct determination of specimen-specific sequences of the hemagglutinin and neuraminidase genes. The two negative control specimens, USDA_13 and USDA_16, demonstrated the capability of the RPM-Flu assay to identify alternative pathogens to avian influenza virus as components of a more complete differential diagnosis assay for cases of avian influenza-like illness.

### Prediction of Influenza Virus A/HN Subtype by Inference from Database Sequence Records That Are Most Similar to RPM-Flu Assay-Generated M, NS or PB Gene Sequences

The results presented in Table 6 demonstrated 19 of 19 reference strains of type A avian influenza viruses detected and correctly identified by corresponding A/HN subtype, based upon the specific HA and NA gene sequences revealed in the assays of each specimen. The same assays also generated specimen-specific M, NS and/or PB2 gene sequences. We used these results to poll the A/HN subtype(s) of the influenza virus strains corresponding to BLAST-returned sequence records found to be most similar to these relatively conserved non-HA and non-NA gene sequences (Supporting Information - Table S6, Table S7, Table S8).

BLAST analysis of the M gene sequences from the 19 avian influenza specimens returned 79 most similar sequence records, of which 40 (51%) were associated with different A/HN subgroups than determined from the corresponding specimens' HA and NA gene sequences. For five of the 19 specimens there were no most similar M gene sequences returned to match the specimen's actual A/HN subtype (Table S6).

BLAST analysis of the NS gene sequences from the 19 avian influenza specimens returned 63 most similar sequence records, of which 41 (65%) were associated with different A/HN subgroups than determined from the corresponding specimens' HA and NA gene sequences. For five of the 19 specimens there were no most similar NS gene sequences returned to match the specimen's actual A/HN subtype (Table S7).

BLAST analysis of the PB2 gene sequences from the 19 avian influenza specimens returned 58 most similar sequence records, of which 22 (38%) were associated with different A/HN subgroups than determined from the corresponding specimens' HA and NA gene sequences. Nine of the 19 specimens had no most similar PB2 gene sequences from the same A/HN subtype (Table S8).

These results suggest little predicate value of most type A influenza virus M, NS or PB2 sequences for determination of the A/HN subtype of the strain from which these sequences were identified.

We referred earlier to the complementary set of field and clinical samples (isolates, not original specimens) of type A avian influenza viruses provided by NAMRU-3 (Cairo, Egypt) and analyzed at NRL using RPM-Flu [18]. As noted earlier, Table S5 in SUPPLEMENTAL INFORMATION presents the C3 Scores from RPM-Flu assay-generated gene sequences data for these specimens.

Eight samples from this previously referenced set of clinical and field specimen isolates of avian influenza virus represented subtypes A/H10N7 (4), A/H7N7 (2), A/H11 (1) or A/H13 (1). In marked contrast to results from RPM-Flu assay-generated HA and NA gene sequences, there was poor correspondence of the actual specimen A/HN subtypes with the subtypes of sequence records returned from BLAST analysis of the assay-generated M, and NS gene sequences. Of 30 most similar M gene sequence records only 5 were from A/HN subtypes matching the subtypes of the corresponding specimens (Table S6). None of the 11 most similar NS gene sequence records matched the A/HN subtypes of the corresponding specimens (Table S7). There were no PB2 gene sequences reported from assays of these eight (non-A/H5N1) specimens (Table S5).

Twenty-one of the remaining specimens of the set reported by Lin et al [18] were of subtype A/H5N1, and the RPM-Flu assay results for these specimens are also included in Table S1, as C3 Scores for the sequences generated from the corresponding HA5, NA1, M, NS and PB2 gene detector tiles. For these 21 A/H5N1-positive specimens, the BLAST analyses of assay-generated M, NS and PB2 gene sequences exclusively represented subtype A/H5N1 avian influenza viruses, as 255 M gene, 146 NS gene and 310 PB2 gene sequence records.

This result for A/H5N1strains is reminiscent of the results presented earlier for subtypes A/H1N1 and A/H3N2 and type B human influenza virus sequence records that are found to be most similar to RPM-Flu assay-generated M, NS and PB2 gene sequences from the corresponding types and subtypes. It is interesting to consider that these results may reflect the highly biased over-representation of samples A/H5N1 sequences in the genome sequence database(s), reflecting much more of the natural diversity of non-HA and non-NA gene sequences than in more sparsely sampled (but naturally far more prevalent) avian influenza virus subtypes.

## Discussion

Rapid and cost-effective microarray-based resequencing (RPM) of influenza virus hemagglutinin and neuraminidase genes provides sensitive detection and specific identification for types and subtypes of previously known as well as emergent strains and variants. These capabilities of the RPM platform stand out very favorably on comparisons with the most recently adopted diagnostic methodologies that are based on real-time reverse-transcription, polymerase chain reaction methods (RT-PCR). The FDA-cleared CDC rRT-PCR Flu Panel (510(k) 080570) enables detection and discrimination of influenza viruses having HA subtype components A/H1, A/H3 and A/H5, and also type B influenza virus. However the test provides no assurance that a positive test result correctly identifies one of the inferred target subtypes A/H1N1, A/H3N2 or A/H5N1; there is no neuraminidase subtype component evaluated as part of the test. Results presented in this report demonstrate that the TessArray RPM-Flu detect and identifies these three targeted subtypes of interest, but also directly distinguishes each of them among the 9 different A/HN subtype categories, or from the background of 144 combinatorial variations of influenza virus A/HN subtypes.

### Sensitivity

The RPM-Flu assay leverages a multiplexed RT-PCR-like strategy to rapidly (∼1 hour) amplify trace quantities of specific target gene sequences so that they may be detected if actually present in a specimen. Results from this report demonstrated that the 95% endpoint limits of detection (LoD) for seasonal A/California-NHRC/BRD10622N/2009(H1N1) and seasonal A/California-NHRC/BRD10601N/2009(H3N2) influenza virus strains were 2.8 infectious doses (TCID_50_) and 5.8 TCID_50_, respectively. These strains have not been serially passaged at high multiplicity in either egg or MDCK cell cultures, so it is not likely that accumulations of defective viral particles would favorably bias determination of sensitivity with genome-based assays relative to infectious doses. Furthermore, these two strains were not selected *a priori* for favorable match of viral sequences with oligonucleotide primers used for target gene sequence amplification. The observed RPM-Flu LoD for each strain is of similar order of magnitude as comparable estimates of analytical sensitivity reported from RT-PCR based methods, Table 7.

**Table 7:** Similar analytical sensitivity for seasonal influenza virus detection using RPM-Flu and rRT-PCR panel assays.

Platform	Tested Strains	Limit of Detection (LoD)
Resequencing Microarray:		
RPM-Flu	A/California-NHRC/BRD10622N/2009(H1N1)	3 TCID_50_
RPM-Flu	A/California-NHRC/BRD10601N/2009(H3N2)	6 TCID_50_
rRT-PCR Panel:		
JBAIDS^a^	A/Hawaii/15/2001 (H1N1)	2 TCID_50_
JBAIDS^a^	A/Texas/71/2007 (H3N2)	15 TCID_50_
CDC^a^	A/New Caledonia/20/1999(H1N1)	16 EID_50_∼1 TCID_50_
CDC^a^	A/Hawaii/15/2001(H1N1)	32 EID_50_∼2 TCID_50_
CDC^a^	A/New York/55/2004(H3N2)	160 EID_50_∼10 TCID_50_
CDC^a^	A/Wisconsin/55/2004(H3N2)	32 EID_50_∼2 TCID_50_

The Limit of Detection (LoD) for seasonal A/H1N1 and seasonal A/H3N2 influenza viruses are presented as the number of tissue culture (TCID_50_) or egg culture (EID_50_) infectious units used for each assay that are demonstrated to result in >95% positive assay outcomes. The relation of tissue culture and egg culture infectious units typically vary with respect to specific influenza virus strain and inoculum. For this table an estimated ratio of 16 EID_50_ : 1 TCID_50_ is based upon results of testing the same A/Hawaii/15/2001 (H1N1) strain by both the JBAIDS and CDC rRT-PCR panels.

a The recently FDA-cleared 510(k)080570 the “CDC Human Influenza Virus Real-time RT-PCR Detection and Characterization Panel” is a test designed to specifically detect contemporary A/H1, A/H3 and A/H5 (Asian Lineage) influenza viruses in humans, as accessed 02 Oct 2009 at http://www.accessdata.fda.gov/cdrh_docs/pdf8/k080570.pdf.

In characterizations of analytical sensitivity of this test panel, the CDC has reported analytical LoD in terms of egg culture infectivity titers (EID_50_/ml). The Department of Defense Joint Biological Agent Identification & Diagnostic System (JBAIDS) has implemented the same RT-PCR test and reported LoD in TCID_50_ units of cell culture infectivity, as accessed 02 Oct 2009 at http://www.fda.gov/downloads/MedicalDevices/Safety/EmergencySituations/UCM180067.pdf.

We estimate CDC rRT-PCR LoD results shown in Table 7 using TCID_50_ units based on the results of both CDC and JBAIDS for A/Hawaii/15/2001(H1N1).

### Specificity

Each RPM assay of a single specimen is capable of generation from hundreds to thousands of nucleotides of multiple targeted pathogen gene sequence(s). Gene sequences generated directly from RPM-Flu assays may specify determinants of particular target pathogen phenotypes (virulence, drug resistance, host range, etc.), or support forensic queries of identity of detected strains in different specimens, or support genome diversity-based epidemiological tracking in the course of an infectious disease outbreak.

RPM-Flu assay-generated nucleotide sequences representing one or more target genes of each detected pathogen convey significantly more informative specificity than can be obtained from any singlet or multiples of RT-PCR tests. Some RT-PCR protocols are designed to glean additional detail regarding detected pathogen genotypes, through results from multiple (serially executed) RT-PCR panels, but this approach imposes additional testing costs and time delays and yield far less specific information than actual target gene sequences provided from a single RPM assay.

Some platforms claim to differentiate selected type A influenza virus subtypes based upon indirect detection or specification of non-HA and non-NA viral gene sequences [21], [22]. Results in this report of RPM-Flu assay-generated viral gene sequences from a variety of reference strains indicate that matrix M gene sequences are correlated with the subtypes of only seasonal human A/H1N1 and seasonal A/H3N2 strains. In general, however, non-HA and non-NA viral gene sequences do not support reliable inference of type A influenza virus subtype.

### Multiplicity

The RPM-Flu assay panel simultaneously addresses 30 different categories of viruses and bacteria that are more or less likely to be encountered in specimens related to respiratory infections (Figure 1, Figure S1). Each assay simultaneously enables detection and identification of targeted pathogens from one or more of 188 different target gene resequencing detector tiles.

For comparison, the recently FDA-cleared Luminex® Respiratory Viral Panel (RVP) Multiplex Nucleic Acid Detection Assay Respiratory Virus Panel (510(k) K063765) is a twelve-plex RT-PCR panel for detection and differentiation of a few strains and types of respiratory viruses including adenovirus, influenza A virus (as A/H1 or A/H3), respiratory syncytial virus, parainfluenza virus, metapneumovirus and rhinovirus (no bacterial respiratory pathogens tested). The RVP assay yields no specimen-specific viral gene sequences that could be used for more detailed identification and differentiation of detected virus strains and variants of detected virus(es).

For human and avian type A influenza viruses in particular, the RPM-Flu provides direct detection and identification capability for any of the combinatorial 144 A/HN subtypes that may be present in a specimen, using an ensemble of 39 different influenza virus target gene resequencing detectors. The Luminex RVP multiplex RT-PCR panel uses three of its twelve primer-probe sets to detect type A influenza virus (M gene-specific primer pair) and distinguish A/H1 from A/H3 (HA1- and HA3-specific primer pairs). While this RT-PCR panel may infer influenza virus subtype, not even testing for the NA component, the assay result is only based on a pattern of signals from a small panel of RT-PCR primer-probes.

Some RT-PCR protocols require serial analysis with hierarchical panels of primer-probes in order to expand the practical multiplicity of pathogen targets or variants. This is the case with the benchmark PCR testing used in this report –a type A influenza virus-positive specimen may be identified in a first round RT-PCR panel, followed by testing with another panel to distinguish A/H1 from A/H3. Failure to establish subtype A/H1 or A/H3 in the follow-on test is a first tier screening results for possible identification of the 2009 Novel A/H1N1 influenza virus outbreak strain. According to the *FACT SHEET FOR HEALTHCARE PROVIDERS: INTERPRETING* CDC *HUMAN INFLUENZA VIRUS REAL-TIME RT-PCR DETECTION AND CHARACTERIZATION PANEL FOR RESPIRATORY SPECIMENS (NPS, NS, TS, NPS/TS, NA1) AND VIRAL CULTURE TEST RESULTS* (Authorized by FDA 02 May 2009),

“The rRT-PCR Flu Panel (**NPS, NS, TS, NPS/TS, NA**) should be ordered to diagnose influenza A infections caused by influenza A or B and subtype determination of seasonal human influenza A virus (seasonal A/H1 or A/H3) to be used as a first tier test for the *in vitro* qualitative detection of novel influenza A (H1N1) virus in patients suspected of having novel influenza A (H1N1) infection. If the test result is positive for influenza A and negative for seasonal H1 and H3 subtypes, the laboratory should test the specimen with the Swine Influenza Real-time RT-PCR Detection Panel (rRT-PCR Swine Flu Panel) for the presumptive detection of novel influenza A (H1N1) infection.”

It is not at all clear that such hierarchical RT-PCR protocols, attempting to increase analytical multiplicity through serial testing of the same specimen(s) at different laboratory venues over a period likely longer than a single day can be either efficient or cost-effective. Far more diagnostic intelligence about the patient and specimen would be available in first-day same-day results from a single RPM-Flu test of the original specimen.

### Utility

Influenza infections do not necessarily preclude co-infection of the same individual by other viral and/or bacterial pathogens (including other strains of influenza virus). Such co-infections may confound a simple diagnosis of influenza, and may also compound morbidity and mortality for a co-infected patient. However it is not common practice that a positive result from a routine diagnostic influenza test would be followed by a call for one or more tests of possible secondary agents of infection. The single specimen single aliquot RPM-Flu assay simultaneously targets 30 different categories of viruses and bacteria, and these were each selected as a respiratory pathogens reported to cause “flu-like” symptoms at some stage of human infection.

Implementation of a highly multiplexed differential diagnostic assay as RPM-Flu would be of great advantage in anticipation of highly likely future influenza outbreaks, epidemics and pandemics. For many decades, leaders in the area of influenza epidemic and pandemic risk assessment and outbreak management have reviewed the pathology of influenza with respect to rapid deterioration and patient deaths attributable to secondary bacterial infections and pneumonia [23], [24].

It has taken almost twenty-five years from the first research reports of the polymerase chain reaction [25] until recent regulatory clearances have enabled introduction specific PCR- and RT-PCR-based diagnostic assays and instrumentation into clinical practice. Such a traditional regulatory clearance timeline may assure safety and efficacy of diagnostic devices beyond imaginable limits of liability, but it also assures that best practice standards for clinical diagnostics are constrained to technologies that perform with nearly obsolete capabilities and specifications. Within the last decade resequencing microarrays have been introduced for research applications and clinical research [26], [27], [28]. It would be disappointing to anticipate that another decade or more may pass before benefits from diagnostic implementations of RPM or other highly multiplexed sequencing-based platforms can be realized in routine clinical diagnostics.

## Supporting Information

Table S1 Analysis of 2009–2010 FluMist live virus trivalent vaccine as comparisons of Influenza type A and type B detector tile sequences to detection and identification of RPM-Flu assay-generated gene sequences. The strains configured in this vaccine are A/South Dakota/06/2007(H1N1), A/Uruguay/716/2007(H3N2) and B/Brisbane/60/2008. The vaccine matrix genes and other non-HA, non-NA genes are from master donor strains A/Ann Arbor/6/1960(H2N2) and B/Ann Arbor/1/66.
(0.06 MB DOC)

Table S2 Analysis of 2009–2010 Fluvirin inactivated virus trivalent vaccine as comparisons of Influenza type A and type B detector tile sequences to detection and identification of RPM-Flu assay-generated gene sequences. The strains configured in this vaccine are A/Brisbane/59/2007, IVR-148 (H1N1), A/Uruguay/716/2007(H3N2) and B/Brisbane/60/2008. The type A virus subtypes of the inactivated vaccines have matrix genes and other non-HA, non-NA genes derived from the master donor strain A/Puerto Rico/8/1934 (H1N1).
(0.07 MB DOC)

Table S3 Analysis of 2005–2006 inactivated virus trivalent vaccine as comparisons of Influenza type A and type B detector tile sequences to detection and identification of RPM-Flu assay-generated gene sequences. The strains configured in this vaccine are A/New Caledonia/20/99 (H1N1), A/New York/55/2004(H3N2) and B/Jiangsu/10/2003. The type A virus subtypes of the inactivated vaccines have matrix genes and other non-HA, non-NA genes derived from the master donor strain A/Puerto Rico/8/1934 (H1N1). RPM-Flu detector title prototype sequences C3 Score BLAST E-value SNPsa Most similar sequence records from BLAST/GenBank include: Hemagglutinin genes A/New Caldedonia/20/1999 (H1N1) 62.3 1e-180 4/934 A/New Caldedonia/20/1999 A/Canterbury/125/2005 (H3N2) 84.6 1e-180 11/1269 A/New York/55/2004 B/Malaysia/2506/2004 25.2 2e-89 23/226 B/Jilin/20/2003 b B/Shanghai/361/2002 55.8 1e-180 20/502 B/Jiangsu/10/2003 b Neuraminidase genes A/New Caldedonia/20/1999 (H1N1) 51.6 1e-180 5/619 A/New Caldedonia/20/1999 A/Canterbury/125/2005 (H3N2) 89.8 1e-180 2/1077 A/New York/55/2004 B/Malaysia/2506/2004 55.2 1e-180 21/662 B/Jiangsu/10/2003 Matrix genes A/Canterbury/100/2000 (H1N1) 54.1 1e-180 24/459 A/Puerto Rico/8/1934(H1N1) A/Canterbury/125/2005 (H3N2) 52.4 1e-180 29/445 A/Puerto Rico/8/1934(H1N1) B/Memphis/13/2003 91.6 1e-180 14/870 B/Jiangsu/10/2003 a SNPs are single base call discrepancies between detector tile sequence and assay generated sequence from labeled target DNA. The number of detected SNPs is shown relative to the number of bases called from the detector tile as contiguous runs of three or more base calls. b The B/Jilin/20/2003 and B/Jiangsu/10/2003 strains are equivalent B/Shanghai/361/2002-like strains as also used in 2004–2005 vaccine configurations (see Table 1).
(0.06 MB DOC)

Table S4 Analysis of 2006–2007 inactivated virus trivalent vaccine as comparisons of Influenza type A and type B detector tile sequences to detection and identification of RPM-Flu assay-generated gene sequences. The strains configured in this vaccine are A/New Caledonia/20/99 (H1N1), A/Wisconsin/67/2005(H3N2) and B/Malaysia/2506/2004. The type A virus subtypes of the inactivated vaccines have matrix genes and other non-HA, non-NA genes derived from the master donor strain A/Puerto Rico/8/1934 (H1N1).
(0.06 MB DOC)

Table S5 RPM-Flu assay detection and identification of avian influenza viruses in field and clinical specimens obtained from human and avian hosts.
(0.11 MB DOC)

Table S6 The A/HN subtypes associated with most similar sequence records for RPM-Flu assay-generated M gene sequences from 19 type A avian influenza viruses are not reliable indicators of the actual A/HN subtype. Forty of 79 (51%) most similar M gene sequence records are associated with different A/HN subtypes than independently determined from each specimen's specific HA and NA gene sequences (mismatches for 8 of 19 specimens).
(0.07 MB DOC)

Table S7 The A/HN subtypes associated with most similar sequence records for RPM-Flu assay-generated NS gene sequences from 19 type A avian influenza viruses are not reliable indicators of the actual A/HN subtype. Forty-one of 63 (65%) most similar NS gene sequence records are associated with different A/HN subtypes than independently determined from each specimen's specific HA and NA gene sequences (mismatches for 11 of 19 specimens).
(0.07 MB DOC)

Table S8 The A/HN subtypes associated with most similar sequence records for RPM-Flu assay-generated PB2 gene sequences from 19 type A avian influenza viruses are not reliable indicators of the actual A/HN subtype. Twenty-two of 58 (38%) most similar PB2 gene sequence records are associated with different A/HN subtypes than independently determined from each specimen's specific HA and NA gene sequences (mismatches for 12 of 19 specimens).
(0.07 MB DOC)

Figure S1 All combinatorial subtypes of type A influenza virus strains and 30 different viral and bacterial pathogens causing influenza-like illness as represented on the RPM-Flu 3.1 resequencing pathogen microarray.
(0.49 MB TIF)
