# Supplementary material for: Single Assay for Simultaneous Detection and Differential Identification of Human and Avian Influenza Virus Types, Subtypes, and Emergent Variants
Source: PLoS One. 2010 Feb 3;5(2):e8995. doi: 10.1371/journal.pone.0008995 (PMC2815781; doi:10.1371/journal.pone.0008995)
Supplement: Table S1 — Analysis of 2009–2010 FluMist live virus trivalent vaccine as comparisons of Influenza type A and type B detector tile sequences to detection and identification of RPM-Flu assay-generated gene sequences. The strains configured in this vaccine are A/South Dakota/06/2007(H1N1), A/Uruguay/716/2007(H3N2) and B/Brisbane/60/2008. The vaccine matrix genes and other non-HA, non-NA genes are from master donor strains A/Ann Arbor/6/1960(H2N2) and B/Ann Arbor/1/66. (0.06 MB DOC) [file pone.0008995.s001.doc]

**Table S1. Analysis of 2009-2010 FluMist live virus trivalent vaccine as comparisons of Influenza type A and type B detector tile sequences to detection and identification of RPM-Flu assay-generated gene sequences. The strains configured in this vaccine are A/South Dakota/06/2007(H1N1), A/Uruguay/716/2007(H3N2) and B/Brisbane/60/2008. The vaccine matrix genes and other non-HA, non-NA genes are from master donor strains A/Ann Arbor/6/1960(H2N2) and B/Ann Arbor/1/66.**

| **RPM-Flu detector title prototype sequences** | **C3**  **Score** | **BLAST**  **E-value** | **SNPs^a^** | **Most similar sequence records from BLAST/GenBank include:** |
| --- | --- | --- | --- | --- |
|  |  |  |  |  |
| **Hemagglutinin genes** |  |  |  |  |
| **A/New Caldedonia/20/1999 (H1N1)** | **84.5** | **1e-180** | **40/1267** | **A/South Dakota/06/2007(H1N1)** |
| **A/Canterbury/125/2005 (H3N2)** | **94.3** | **1e-180** | **17/1414** | **A/Uruguay/716/2007(H3N2)** |
| **B/Malaysia/2506/2004** | **94.9** | **1e-180** | **12/854** | **B/Brisbane/60/2008** |
| **B/Shanghai/361/2002** | **51.4** | **1e-180** | **46/462** | **B/Brisbane/60/2008** |
|  |  |  |  |  |
| **Neuraminidase genes** |  |  |  |  |
| **A/New Caldedonia/20/1999 (H1N1)** | **93.8** | **1e-180** | **27/1125** | **A/South Dakota/06/2007(H1N1)** |
| **A/Canterbury/125/2005 (H3N2)** | **95.9** | **1e-180** | **12/1150** | **A/Uruguay/716/2007(H3N2)** |
| **B/Malaysia/2506/2004** | **91.7** | **1e-180** | **24/1100** | **B/Brisbane/60/2008** |
|  |  |  |  |  |
| **Matrix genes** |  |  |  |  |
| **A/Canterbury/100/2000 (H1N1)** | **78.8** | **1e-180** | **27/669** | **A/Ann Arbor/6/1960(H2N2)** |
| **A/Canterbury/125/2005 (H3N2)** | **82.0** | **1e-180** | **35/697** | **A/Ann Arbor/6/1960(H2N2)** |
| **B/Memphis/13/2003** | **86.8** | **1e-180** | **30/824** | **B/Ann Arbor/1/66** |

**^a^ SNPs are single base call discrepancies between detector tile sequence and assay generated sequence from labeled target DNA. The number of detected SNPs is shown relative to the number of bases called from the detector tile as contiguous runs of three or more base calls.**
