# Supplementary material for: Single Assay for Simultaneous Detection and Differential Identification of Human and Avian Influenza Virus Types, Subtypes, and Emergent Variants
Source: PLoS One. 2010 Feb 3;5(2):e8995. doi: 10.1371/journal.pone.0008995 (PMC2815781; doi:10.1371/journal.pone.0008995)
Supplement: Table S2 — Analysis of 2009–2010 Fluvirin inactivated virus trivalent vaccine as comparisons of Influenza type A and type B detector tile sequences to detection and identification of RPM-Flu assay-generated gene sequences. The strains configured in this vaccine are A/Brisbane/59/2007, IVR-148 (H1N1), A/Uruguay/716/2007(H3N2) and B/Brisbane/60/2008. The type A virus subtypes of the inactivated vaccines have matrix genes and other non-HA, non-NA genes derived from the master donor strain A/Puerto Rico/8/1934 (H1N1). (0.07 MB DOC) [file pone.0008995.s002.doc]

**Table S2. Analysis of 2009-2010 Fluvirin inactivated virus trivalent vaccine as comparisons of Influenza type A and type B detector tile sequences to detection and identification of RPM-Flu assay-generated gene sequences. The strains configured in this vaccine are A/Brisbane/59/2007, IVR-148 (H1N1), A/Uruguay/716/2007(H3N2) and B/Brisbane/60/2008. The type A virus subtypes of the inactivated vaccines have matrix genes and other non-HA, non-NA genes derived from the master donor strain A/Puerto Rico/8/1934 (H1N1).**

| **RPM-Flu detector title prototype sequences** | **C3**  **Score** | **BLAST**  **E-value** | **SNPs^a^** | **Most similar sequence records from BLAST/GenBank include:** |
| --- | --- | --- | --- | --- |
|  |  |  |  |  |
| **Hemagglutinin genes** |  |  |  |  |
| **A/New Caldedonia/20/1999 (H1N1)** | **35.7** | **1e-180** | **26/535** | **A/Brisbane/59/2007** |
| **A/Canterbury/125/2005 (H3N2)** | **58.5** | **1e-180** | **15/877** | **A/Uruguay/716/2007(H3N2)** |
| **B/Malaysia/2506/2004** | **32.0** | **2e-116** | **15/288** | **B/Brisbane/60/2008** |
| **B/Shanghai/361/2002** | **14.7** | **9e-51** | **39/132** | **B/Brisbane/60/2008** |
|  |  |  |  |  |
| **Neuraminidase genes** |  |  |  |  |
| **A/New Caldedonia/20/1999 (H1N1)** | **24.0** | **3e-121** | **28/288** | **A/Brisbane/59/2007** |
| **A/Canterbury/125/2005 (H3N2)** | **51.0** | **1e-180** | **13/612** | **A/Uruguay/716/2007(H3N2)** |
| **B/Malaysia/2506/2004** | **14.7** | **9e-51** | **31/176** | **B/Brisbane/60/2008** |
|  |  |  |  |  |
| **Matrix genes** |  |  |  |  |
| **A/Canterbury/100/2000 (H1N1)** | **46.0** | **6e-161** | **27/391** | **A/Puerto Rico/8/1934(H1N1)** |
| **A/Canterbury/125/2005 (H3N2)** | **42.3** | **7e-165** | **32/359** | **A/Puerto Rico/8/1934(H1N1)** |
| **B/Memphis/13/2003** | **39.6** | **5e-163** | **5/376** | **[B/Taiwan/1734/2006] ^b^** |

**^a^ SNPs are single base call discrepancies between detector tile sequence and assay generated sequence from labeled target DNA. The number of detected SNPs is shown relative to the number of bases called from the detector tile as contiguous runs of three or more base calls.**

**^b^ No matrix gene (segment 7) sequence of the vaccine strain B/Brisbane/60/2008 is in accession at GenBank (as of 15 Dec 2009), nor yet included in the TessArray RPM VSRD. It is likely that the most similar sequence record matching the type B influenza virus matrix gene component of the trivalent vaccine is identical or nearly identical to the actual matrix sequence of B/Brisbane/60/2008. A similar situation arose with delayed accession of neuraminidase gene sequences from B/Jilin/20/2003 used in the 2004-2005 live viral FluMist vaccine configuration (See Table 1).**
