# Supplementary material for: Single Assay for Simultaneous Detection and Differential Identification of Human and Avian Influenza Virus Types, Subtypes, and Emergent Variants
Source: PLoS One. 2010 Feb 3;5(2):e8995. doi: 10.1371/journal.pone.0008995 (PMC2815781; doi:10.1371/journal.pone.0008995)
Supplement: Table S3 — Analysis of 2005–2006 inactivated virus trivalent vaccine as comparisons of Influenza type A and type B detector tile sequences to detection and identification of RPM-Flu assay-generated gene sequences. The strains configured in this vaccine are A/New Caledonia/20/99 (H1N1), A/New York/55/2004(H3N2) and B/Jiangsu/10/2003. The type A virus subtypes of the inactivated vaccines have matrix genes and other non-HA, non-NA genes derived from the master donor strain A/Puerto Rico/8/1934 (H1N1). RPM-Flu detector title prototype sequences C3 Score BLAST E-value SNPsa Most similar sequence records from BLAST/GenBank include: Hemagglutinin genes A/New Caldedonia/20/1999 (H1N1) 62.3 1e-180 4/934 A/New Caldedonia/20/1999 A/Canterbury/125/2005 (H3N2) 84.6 1e-180 11/1269 A/New York/55/2004 B/Malaysia/2506/2004 25.2 2e-89 23/226 B/Jilin/20/2003 b B/Shanghai/361/2002 55.8 1e-180 20/502 B/Jiangsu/10/2003 b Neuraminidase genes A/New Caldedonia/20/1999 (H1N1) 51.6 1e-180 5/619 A/New Caldedonia/20/1999 A/Canterbury/125/2005 (H3N2) 89.8 1e-180 2/1077 A/New York/55/2004 B/Malaysia/2506/2004 55.2 1e-180 21/662 B/Jiangsu/10/2003 Matrix genes A/Canterbury/100/2000 (H1N1) 54.1 1e-180 24/459 A/Puerto Rico/8/1934(H1N1) A/Canterbury/125/2005 (H3N2) 52.4 1e-180 29/445 A/Puerto Rico/8/1934(H1N1) B/Memphis/13/2003 91.6 1e-180 14/870 B/Jiangsu/10/2003 a SNPs are single base call discrepancies between detector tile sequence and assay generated sequence from labeled target DNA. The number of detected SNPs is shown relative to the number of bases called from the detector tile as contiguous runs of three or more base calls. b The B/Jilin/20/2003 and B/Jiangsu/10/2003 strains are equivalent B/Shanghai/361/2002-like strains as also used in 2004–2005 vaccine configurations (see Table 1). (0.06 MB DOC) [file pone.0008995.s003.doc]

**Table S3. Analysis of 2005-2006 inactivated virus trivalent vaccine as comparisons of Influenza type A and type B detector tile sequences to detection and identification of RPM-Flu assay-generated gene sequences. The strains configured in this vaccine are A/New Caledonia/20/99 (H1N1), A/New York/55/2004(H3N2) and B/Jiangsu/10/2003. The type A virus subtypes of the inactivated vaccines have matrix genes and other non-HA, non-NA genes derived from the master donor strain A/Puerto Rico/8/1934 (H1N1).**

| **RPM-Flu detector title prototype sequences** | **C3**  **Score** | **BLAST**  **E-value** | **SNPs^a^** | **Most similar sequence records from BLAST/GenBank include:** |
| --- | --- | --- | --- | --- |
|  |  |  |  |  |
| **Hemagglutinin genes** |  |  |  |  |
| **A/New Caldedonia/20/1999 (H1N1)** | **62.3** | **1e-180** | **4/934** | **A/New Caldedonia/20/1999** |
| **A/Canterbury/125/2005 (H3N2)** | **84.6** | **1e-180** | **11/1269** | **A/New York/55/2004** |
| **B/Malaysia/2506/2004** | **25.2** | **2e-89** | **23/226** | **B/Jilin/20/2003 ^b^** |
| **B/Shanghai/361/2002** | **55.8** | **1e-180** | **20/502** | **B/Jiangsu/10/2003 ^b^** |
|  |  |  |  |  |
| **Neuraminidase genes** |  |  |  |  |
| **A/New Caldedonia/20/1999 (H1N1)** | **51.6** | **1e-180** | **5/619** | **A/New Caldedonia/20/1999** |
| **A/Canterbury/125/2005 (H3N2)** | **89.8** | **1e-180** | **2/1077** | **A/New York/55/2004** |
| **B/Malaysia/2506/2004** | **55.2** | **1e-180** | **21/662** | **B/Jiangsu/10/2003** |
|  |  |  |  |  |
| **Matrix genes** |  |  |  |  |
| **A/Canterbury/100/2000 (H1N1)** | **54.1** | **1e-180** | **24/459** | **A/Puerto Rico/8/1934(H1N1)** |
| **A/Canterbury/125/2005 (H3N2)** | **52.4** | **1e-180** | **29/445** | **A/Puerto Rico/8/1934(H1N1)** |
| **B/Memphis/13/2003** | **91.6** | **1e-180** | **14/870** | **B/Jiangsu/10/2003** |

**^a^ SNPs are single base call discrepancies between detector tile sequence and assay generated sequence from labeled target DNA. The number of detected SNPs is shown relative to the number of bases called from the detector tile as contiguous runs of three or more base calls.**

**^b^ The B/Jilin/20/2003 and B/Jiangsu/10/2003 strains are equivalent B/Shanghai/361/2002-like strains as also used in 2004-2005 vaccine configurations (see Table 1).**
