# Supplementary material for: Single Assay for Simultaneous Detection and Differential Identification of Human and Avian Influenza Virus Types, Subtypes, and Emergent Variants
Source: PLoS One. 2010 Feb 3;5(2):e8995. doi: 10.1371/journal.pone.0008995 (PMC2815781; doi:10.1371/journal.pone.0008995)
Supplement: Table S4 — Analysis of 2006–2007 inactivated virus trivalent vaccine as comparisons of Influenza type A and type B detector tile sequences to detection and identification of RPM-Flu assay-generated gene sequences. The strains configured in this vaccine are A/New Caledonia/20/99 (H1N1), A/Wisconsin/67/2005(H3N2) and B/Malaysia/2506/2004. The type A virus subtypes of the inactivated vaccines have matrix genes and other non-HA, non-NA genes derived from the master donor strain A/Puerto Rico/8/1934 (H1N1). (0.06 MB DOC) [file pone.0008995.s004.doc]

**Table S4. Analysis of 2006-2007 inactivated virus trivalent vaccine as comparisons of Influenza type A and type B detector tile sequences to detection and identification of RPM-Flu assay-generated gene sequences. The strains configured in this vaccine are A/New Caledonia/20/99 (H1N1), A/Wisconsin/67/2005(H3N2) and B/Malaysia/2506/2004. The type A virus subtypes of the inactivated vaccines have matrix genes and other non-HA, non-NA genes derived from the master donor strain A/Puerto Rico/8/1934 (H1N1).**

| **RPM-Flu detector title prototype sequences** | **C3**  **Score** | **BLAST**  **E-value** | **SNPs^a^** | **Most similar sequence records from BLAST/GenBank include:** |
| --- | --- | --- | --- | --- |
|  |  |  |  |  |
| **Hemagglutinin genes** |  |  |  |  |
| **A/New Caldedonia/20/1999 (H1N1)** | **91.4** | **1e-180** | **2/1371** | **A/New Caldedonia/20/1999** |
| **A/Canterbury/125/2005 (H3N2)** | **94.3** | **1e-180** | **7/1414** | **A/Wisconsin/67/2005** |
| **B/Malaysia/2506/2004** | **95.8** | **1e-180** | **1/862** | **B/Malaysia/2506/2004** |
| **B/Shanghai/361/2002** | **36.9** | **1e-180** | **46/332** | **B/Malaysia/2506/2004** |
|  |  |  |  |  |
| **Neuraminidase genes** |  |  |  |  |
| **A/New Caldedonia/20/1999 (H1N1)** | **87.8** | **1e-180** | **5/1053** | **A/New Caldedonia/20/1999** |
| **A/Canterbury/125/2005 (H3N2)** | **96.8** | **1e-180** | **2/1161** | **A/Wisconsin/67/2005** |
| **B/Malaysia/2506/2004** | **98.0** | **1e-180** | **1/1176** | **B/Malaysia/2506/2004** |
|  |  |  |  |  |
| **Matrix genes** |  |  |  |  |
| **A/Canterbury/100/2000 (H1N1)** | **61.6** | **1e-180** | **35523** | **A/Puerto Rico/8/1934(H1N1)** |
| **A/Canterbury/125/2005 (H3N2)** | **59.2** | **1e-180** | **39/503** | **A/Puerto Rico/8/1934(H1N1)** |
| **B/Memphis/13/2003** | **97.7** | **1e-180** | **6/928** | **B/Malaysia/2506/2004** |

**^a^ SNPs are single base call discrepancies between detector tile sequence and assay generated sequence from labeled target DNA. The number of detected SNPs is shown relative to the number of bases called from the detector tile as contiguous runs of three or more base calls.**
