# Supplementary material for: Single Assay for Simultaneous Detection and Differential Identification of Human and Avian Influenza Virus Types, Subtypes, and Emergent Variants
Source: PLoS One. 2010 Feb 3;5(2):e8995. doi: 10.1371/journal.pone.0008995 (PMC2815781; doi:10.1371/journal.pone.0008995)
Supplement: Table S5 — RPM-Flu assay detection and identification of avian influenza viruses in field and clinical specimens obtained from human and avian hosts. (0.11 MB DOC) [file pone.0008995.s005.doc]

**Table S5. RPM-Flu Assay Detection and Identification of Avian Influenza Viruses in Field and Clinical Specimens obtained from Human and Avian Hosts**

| **Specimen** | **Detection**  **Subtype** | **C3 HA**  **(by Subtype)** | **C3 NA**  **(by Subtype)** | **C3 M**  **(H5N1)** | **C3 NS**  **(H5N1)** | **C3 PB2**  **(H5N1)** |
| --- | --- | --- | --- | --- | --- | --- |
| **Human Host** |  |  |  |  |  |  |
| **900755_2006** | **H5N1** | **66.9** | **62.6** | **83.5** | **79.1** | **77.9** |
| **902786_2006** | **H5N1** | **52.4** | **39.7** | **81.9** | **75.2** | **69.8** |
| **902834_2006** | **H5N1** | **37.0** | **44.9** | **71.7** | **74.1** | **72.2** |
| **902838_2006** | **H5N1** | **25.3** | **70.0** | **76.4** | **74.5** | **19.1** |
| **902782_2006** | **H5N1** | **18.3** | **10.0** | **65.1** | **73.1** | **22.0** |
| **900756_2006** | **H5N1** | **14.8** | **31.1** | **78.9** | **54.8** | **46.1** |
| **903458_2006** | **(H5N1)** | 3.1 | 6.0 | **28.9** | **41.7** | **38.3** |
| **Human Blank** |  |  |  |  |  |  |
| **902947_2006** | **Negative** | 2.4 | 3.4 | 0.4 | 2.5 | 1.4 |
| **Avian Host** |  |  |  |  |  |  |
| **900207_2006** | **H5N1** | **79.1** | **63.1** | **79.5** | **68.8** | **86.1** |
| **900845_2006** | **H5N1** | **76.2** | **61.6** | **69.7** | **73.8** | **79.7** |
| **900590_2006** | **H5N1** | **74.7** | **62.5** | **77.8** | **73.4** | **80.0** |
| **900844_2006** | **H5N1** | **74.3** | **62.3** | **79.8** | **81.2** | **80.6** |
| **900588_2006** | **H5N1** | **70.9** | **57.2** | **71.3** | **75.6** | **80.5** |
| **900839_2006** | **H5N1** | **68.7** | **65.4** | **75.3** | **79.5** | **72.7** |
| **901162_2006** | **H5N1** | **67.8** | **58.0** | **70.6** | **77.4** | **79.2** |
| **902992_2006** | **H5N1** | **64.0** | **49.4** | **84.0** | **78.8** | **76.5** |
| **906375_2006** | **H5N1** | **62.4** | **56.4** | **75.1** | **76.1** | **76.9** |
| **906089_2006** | **H5N1** | **61.0** | **71.9** | **69.6** | **66.1** | **50.6** |
| **909467_2005** | **H5N1** | **58.1** | **90.1** | **82.9** | **85.7** | **11.9** |
| **900840_2006** | **H5N1** | **47.1** | **50.5** | **82.4** | **76.8** | **57.5** |
| **906608_2006** | **H5N1** | **42.8** | **40.8** | **75.1** | **73.3** | **63.9** |
| **902764_2006** | **H5N1** | **40.3** | **84.1** | **88.1** | **85.8** | 9.5 |
| **900843_2006** | **(H5N1)** | 3.2 | 4.6 | **42.3** | 2.1 | 3.6 |
| **902991_2006** | **(H5N1)** | 3.6 | 4.5 | 2.1 | **42.5** | 2.3 |
| **909864_2004** | **H7N7** | **87.3** | **66.7** | **69.9** | **83.3** | 7.0 |
| **905588_2006** | **H7N7** | **53.4** | **47.7** | **49.5** | **65.4** | 4.8 |
| **912908_2005** | **H10N7** | **72.7** | **32.0** | **50.9** | **72.7** | 3.2 |
| **900845_2004** | **H10(N7)** | **66.4** | 3.8 | **48.3** | **71.4** | 1.0 |
| **900600_2004** | **H10N7** | **61.9** | **23.1** | **49.8** | **69.0** | 1.2 |
| **912823_2006** | **H10N7** | **57.2** | **15.8** | **48.9** | **70.5** | 0.6 |
| **900688_2004** | **H11(N?)** | **50.6** | 4.1 | **53.8** | **73.4** | 4.4 |
| **912306_2005** | **H13(N?)** | **37.0** | 1.8 | **34.2** | **19.1** | 1.6 |
| **909888_2005** | **AI** | 3.3 | 7.8 | **65.0** | 4.0 | 2.2 |
| **910801_2005** | **AI** | 7.6 | 2.5 | **20.1** | 1.8 | 0.0 |
| **920431_2003** | **AI** | 1.0 | 1.2 | **13.3** | 7.4 | 0.0 |
| **Avian Blank** |  |  |  |  |  |  |
| **900134_2005** | **Negative** | 3.1 | 3.1 | 1.6 | 2.9 | 0.7 |
| **910809_2005** | **Negative** | 4.4 | 3.7 | 2.9 | 3.9 | 1.2 |
| **910868_2005** | **Negative** | 1.7 | 1.5 | 0.0 | 1.8 | 0.7 |
| **910876_2005** | **Negative** | 2.7 | 3.2 | 0.7 | 2.3 | 1.1 |
| **910878_2005** | **Negative** | 2.4 | 2.8 | 1.3 | 2.3 | 0.9 |
| **917518_2003** | **Negative** | 2.3 | 1.5 | 0.8 | 2.9 | 0.6 |
