# Supplementary material for: Single Assay for Simultaneous Detection and Differential Identification of Human and Avian Influenza Virus Types, Subtypes, and Emergent Variants
Source: PLoS One. 2010 Feb 3;5(2):e8995. doi: 10.1371/journal.pone.0008995 (PMC2815781; doi:10.1371/journal.pone.0008995)
Supplement: Table S6 — The A/HN subtypes associated with most similar sequence records for RPM-Flu assay-generated M gene sequences from 19 type A avian influenza viruses are not reliable indicators of the actual A/HN subtype. Forty of 79 (51%) most similar M gene sequence records are associated with different A/HN subtypes than independently determined from each specimen's specific HA and NA gene sequences (mismatches for 8 of 19 specimens). (0.07 MB DOC) [file pone.0008995.s006.doc]

**Table S6. The A/HN subtypes associated with most similar sequence records for RPM-Flu assay-generated M gene sequences from 19 type A avian influenza viruses are not reliable indicators of the actual A/HN subtype. Forty of 79 (51%) most similar M gene sequence records are associated with different A/HN subtypes than independently determined from each specimen’s specific HA and NA gene sequences (mismatches for 8 of 19 specimens).**

| **M-Gene Targets** | | **MATCH** | **MISMATCH** | **Mismatched A/HN Subtypes from Most Similar M Gene Sequence Records** | | | | | | | | | |
| --- | --- | --- | --- | --- | --- | --- | --- | --- | --- | --- | --- | --- | --- |
| **USDA_1** | **A/H1N1** | **2** | **0** |  |  |  |  |  |  |  |  |  |  |
| **USDA_2** | **A/H2N8** | **2** | **5** | **1-H4N9** | **2-H4N6** | **1-H3N8** | **1-H6N8** |  |  |  |  |  |  |
| **USDA_3** | **A/H3N2** | **2** | **1** | **1-H1N1** |  |  |  |  |  |  |  |  |  |
| **USDA_4** | **A/H4N6** | **1** | **0** |  |  |  |  |  |  |  |  |  |  |
| **USDA_6** | **A/H7N2** | **1** | **0** |  |  |  |  |  |  |  |  |  |  |
| **USDA_7** | **A/H8N4** | **0** | **3** | **1-H3N9** | **1-H6N1** | **1-H6N-** |  |  |  |  |  |  |  |
| **USDA_8** | **A/H11N9** | **0** | **5** | **1-H5N8** | **1-H6N2** | **1-H7N1** | **2-H10N7** |  |  |  |  |  |  |
| **USDA_9** | **A/H10N7** | **0** | **6** | **1-H2N1** | **1-H7N9** | **1-H3N6** | **1-H6N8** | **1-H1N9** | **1-H3N8** |  |  |  |  |
| **USDA_10** | **A/H11N3** | **0** | **3** | **3-H7N2** |  |  |  |  |  |  |  |  |  |
| **USDA_11** | **A/H12N5** | **0** | **12** | **2-H3N8** | **2-H11N2** | **1-H4N6** | **1-H3N4** | **1-H4N8** | **1-H5N1** | **1-H11N9** | **1-H1N9** | **1-H2N3** | **1-H5N2** |
| **USDA_12** | **A/H13N6** | **1** | **1** | **1-H13N9** |  |  |  |  |  |  |  |  |  |
| **USDA_14** | **A/H5N3** | **1** | **0** |  |  |  |  |  |  |  |  |  |  |
| **USDA_15** | **A/H7N3** | **7** | **0** |  |  |  |  |  |  |  |  |  |  |
| **USDA_17** | **A/H5N2** | **3** | **0** |  |  |  |  |  |  |  |  |  |  |
| **USDA_18** | **A/H7N1** | **10** | **0** |  |  |  |  |  |  |  |  |  |  |
| **USDA_19** | **A/H7N3** | **1** | **4** | **1-H11N9** | **1-H5N3** | **1-H1N1** | **1-H5N2** |  |  |  |  |  |  |
| **USDA_20** | **A/H7N7** | **4** | **0** |  |  |  |  |  |  |  |  |  |  |
| **USDA_21** | **A/H14N5** | **1** | **0** |  |  |  |  |  |  |  |  |  |  |
| **USDA_22** | **A/H15N9** | **3** | **0** |  |  |  |  |  |  |  |  |  |  |
