# Supplementary material for: Single Assay for Simultaneous Detection and Differential Identification of Human and Avian Influenza Virus Types, Subtypes, and Emergent Variants
Source: PLoS One. 2010 Feb 3;5(2):e8995. doi: 10.1371/journal.pone.0008995 (PMC2815781; doi:10.1371/journal.pone.0008995)
Supplement: Table S7 — The A/HN subtypes associated with most similar sequence records for RPM-Flu assay-generated NS gene sequences from 19 type A avian influenza viruses are not reliable indicators of the actual A/HN subtype. Forty-one of 63 (65%) most similar NS gene sequence records are associated with different A/HN subtypes than independently determined from each specimen's specific HA and NA gene sequences (mismatches for 11 of 19 specimens). (0.07 MB DOC) [file pone.0008995.s007.doc]

**Table S7. The A/HN subtypes associated with most similar sequence records for RPM-Flu assay-generated NS gene sequences from 19 type A avian influenza viruses are not reliable indicators of the actual A/HN subtype. Forty-one of 63 (65%) most similar NS gene sequence records are associated with different A/HN subtypes than independently determined from each specimen’s specific HA and NA gene sequences (mismatches for 11 of 19 specimens).**

| **NS-Gene Targets** | | **MATCH** | **MISMATCH** | **Mismatched A/HN Subtypes from Most Similar NS Gene Sequence Records** | | | | | | | |
| --- | --- | --- | --- | --- | --- | --- | --- | --- | --- | --- | --- |
| **USDA_1** | **A/H1N1** | **6** | **0** |  |  |  |  |  |  |  |  |
| **USDA_2** | **A/H2N8** | **2** | **8** | **1-**  **H9N5** | **1-H2N9** | **1-H11N1** | **1-H11N4** | **1-H11N9** | **1-H9N1** | **1-H9N5** | **1-H2N1** |
| **USDA_3** | **A/H3N2** | **1** | **0** |  |  |  |  |  |  |  |  |
| **USDA_4** | **A/H4N6** |  | **0** |  |  |  |  |  |  |  |  |
| **USDA_6** | **A/H7N2** | **1** | **1** | **1-**  **H6N8** |  |  |  |  |  |  |  |
| **USDA_7** | **A/H8N4** | **1** | **5** | **1-H10N7** | **1-H5N1** | **1-H11N2** | **1-H5N2** | **1-H8N4** |  |  |  |
| **USDA_8** | **A/H11N9** | **0** | **1** | **1-H2N3** |  |  |  |  |  |  |  |
| **USDA_9** | **A/H10N7** | **1** | **1** | **1-H1N1** |  |  |  |  |  |  |  |
| **USDA_10** | **A/H11N3** |  | **0** |  |  |  |  |  |  |  |  |
| **USDA_11** | **A/H12N5** | **0** | **4** | **1-H5N2** | **2-H4N6** | **1-H10N7** |  |  |  |  |  |
| **USDA_12** | **A/H13N6** | **0** | **1** | **1-H13N9** |  |  |  |  |  |  |  |
| **USDA_14** | **A/H5N3** | **0** | **1** | **1-H5N1** |  |  |  |  |  |  |  |
| **USDA_15** | **A/H7N3** | **0** | **12** | **12-H5N1** |  |  |  |  |  |  |  |
| **USDA_17** | **A/H5N2** |  |  |  |  |  |  |  |  |  |  |
| **USDA_18** | **A/H7N1** |  |  |  |  |  |  |  |  |  |  |
| **USDA_19** | **A/H7N3** | **2** | **0** |  |  |  |  |  |  |  |  |
| **USDA_20** | **A/H7N7** | **5** | **0** |  |  |  |  |  |  |  |  |
| **USDA_21** | **A/H14N5** | **0** | **2** | **2-H5N1** |  |  |  |  |  |  |  |
| **USDA_22** | **A/H15N9** | **3** | **5** | **1-H15N2** | **1-H4N4** | **2-H4N6** | **1-H6N2** |  |  |  |  |
