# Supplementary material for: Single Assay for Simultaneous Detection and Differential Identification of Human and Avian Influenza Virus Types, Subtypes, and Emergent Variants
Source: PLoS One. 2010 Feb 3;5(2):e8995. doi: 10.1371/journal.pone.0008995 (PMC2815781; doi:10.1371/journal.pone.0008995)
Supplement: Table S8 — The A/HN subtypes associated with most similar sequence records for RPM-Flu assay-generated PB2 gene sequences from 19 type A avian influenza viruses are not reliable indicators of the actual A/HN subtype. Twenty-two of 58 (38%) most similar PB2 gene sequence records are associated with different A/HN subtypes than independently determined from each specimen's specific HA and NA gene sequences (mismatches for 12 of 19 specimens). (0.07 MB DOC) [file pone.0008995.s008.doc]

**Table S8. The A/HN subtypes associated with most similar sequence records for RPM-Flu assay-generated PB2 gene sequences from 19 type A avian influenza viruses are not reliable indicators of the actual A/HN subtype. Twenty-two of 58 (38%) most similar PB2 gene sequence records are associated with different A/HN subtypes than independently determined from each specimen’s specific HA and NA gene sequences (mismatches for 12 of 19 specimens).**

| **PB2-Gene Targets** | | **MATCH** | **MISMATCH** | **Mismatched A/HN Subtypes from Most Similar PB2 Gene Sequence Records** | | | | | |
| --- | --- | --- | --- | --- | --- | --- | --- | --- | --- |
| **USDA_1** | **A/H1N1** | **0** | **2** | **1-H3N2** | **1-H9N2** |  |  |  |  |
| **USDA_2** | **A/H2N8** | **3** | **1** | **1-H2N1** |  |  |  |  |  |
| **USDA_3** | **A/H3N2** | **1** | **0** |  |  |  |  |  |  |
| **USDA_4** | **A/H4N6** | **0** | **2** | **2-H1N1** |  |  |  |  |  |
| **USDA_6** | **A/H7N2** | **1** | **1** | **1-H6N8** |  |  |  |  |  |
| **USDA_7** | **A/H8N4** | **0** | **1** | **1H4N6** |  |  |  |  |  |
| **USDA_8** | **A/H11N9** |  | **1** | **1-H2N3** |  |  |  |  |  |
| **USDA_9** | **A/H10N7** | **0** | **1** | **1-H5N2** |  |  |  |  |  |
| **USDA_10** | **A/H11N3** | **0** | **5** | **5-H7N2** |  |  |  |  |  |
| **USDA_11** | **A/H12N5** | **0** | **2** | **2-H1N1** |  |  |  |  |  |
| **USDA_12** | **A/H13N6** | **0** | **2** | **1-H9N2** | **1-H6N5** |  |  |  |  |
| **USDA_14** | **A/H5N3** | **1** | **0** |  |  |  |  |  |  |
| **USDA_15** | **A/H7N3** | **1** | **0** |  |  |  |  |  |  |
| **USDA_17** | **A/H5N2** | **4** | **0** |  |  |  |  |  |  |
| **USDA_18** | **A/H7N1** | **19** | **0** |  |  |  |  |  |  |
| **USDA_19** | **A/H7N3** | **1** | **0** |  |  |  |  |  |  |
| **USDA_20** | **A/H7N7** | **2** | **0** |  |  |  |  |  |  |
| **USDA_21** | **A/H14N5** | **0** | **3** | **3-H5N1** |  |  |  |  |  |
| **USDA_22** | **A/H15N9** | **3** | **1** | **1-H15N2** |  |  |  |  |  |
