# Supplementary figures and images for: Single Assay for Simultaneous Detection and Differential Identification of Human and Avian Influenza Virus Types, Subtypes, and Emergent Variants
Source: PLoS One. 2010 Feb 3;5(2):e8995. doi: 10.1371/journal.pone.0008995 (PMC2815781; doi:10.1371/journal.pone.0008995)

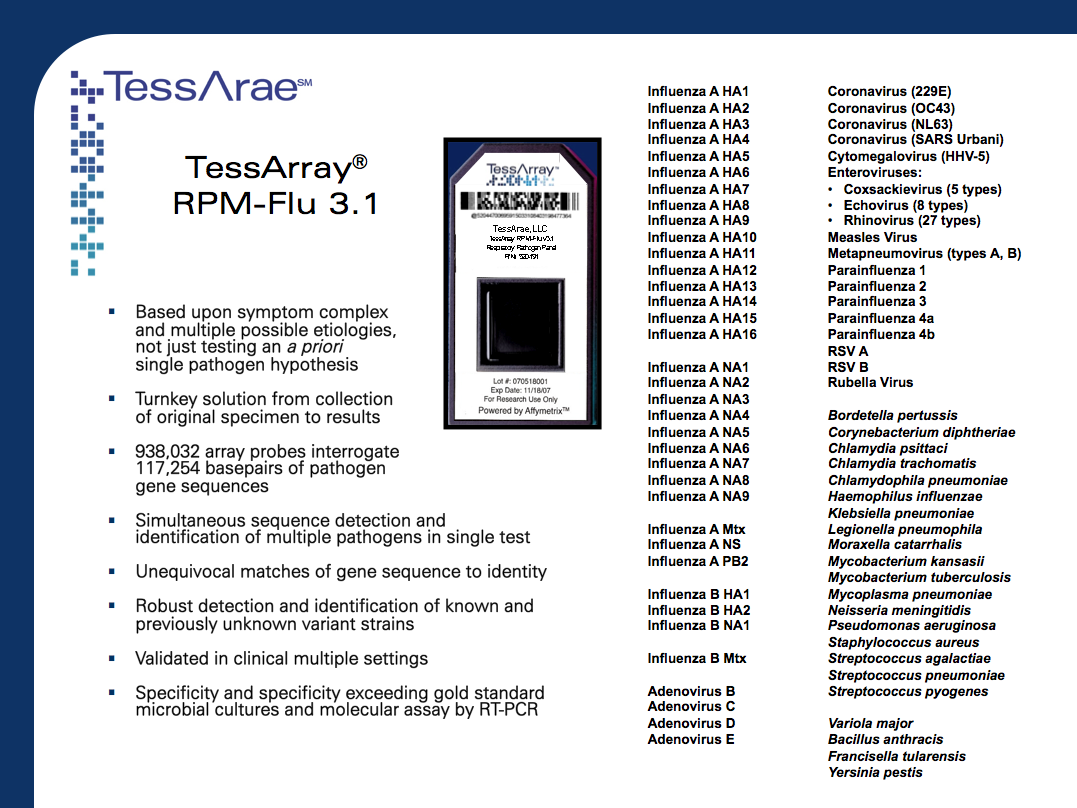

Supplement: Figure S1 — All combinatorial subtypes of type A influenza virus strains and 30 different viral and bacterial pathogens causing influenza-like illness as represented on the RPM-Flu 3.1 resequencing pathogen microarray. (0.49 MB TIF) [file pone.0008995.s009.tif]
